# ^18^F-FSPG PET imaging for the evaluation of indeterminate pulmonary nodules

**DOI:** 10.1371/journal.pone.0265427

**Published:** 2022-03-16

**Authors:** Rafael Paez, Chirayu Shah, Angelina J. Cords, Anel Muterspaugh, John E. Helton, Sanja Antic, Rosana Eisenberg, Heidi Chen, Eric L. Grogan, Henry C. Manning, Ronald C. Walker, Pierre P. Massion

**Affiliations:** 1 Department of Medicine, Division of Allergy, Pulmonary and Critical Care Medicine, Vanderbilt University Medical Center, Nashville, Tennessee, United States of America; 2 Department of Radiology and Radiological Sciences, Vanderbilt University Medical Center, Nashville, Tennessee, United States of America; 3 Veterans Affairs Hospital, Tennessee Valley Healthcare System, Nashville, Tennessee, United States of America; 4 Department of Research Service, Tennessee Healthcare Valley System, Nashville, Tennessee, United States of America; 5 Department of Pathology, Microbiology and Immunology, Vanderbilt University Medical Center, Nashville, Tennessee, United States of America; 6 Department of Biostatistics, Vanderbilt University Medical Center, Nashville, Tennessee, United States of America; 7 Department of Thoracic Surgery, Vanderbilt University Medical Center, Nashville, Tennessee, United States of America; 8 Center for Advanced Biomedical Imaging, Department of Cancer Systems Imaging, The University of Texas MD Anderson Cancer Center, Houston, Texas, United States of America; 9 Cancer Early Detection and Prevention Initiative, Vanderbilt Ingram Cancer Center, Nashville, Tennessee, United States of America; Mie University Graduate School of Medicine, JAPAN

## Abstract

**Background:**

^18^F-fluorodeoxyglucose (FDG) PET/CT is recommended for evaluation of intermediate-risk indeterminate pulmonary nodules (IPNs). While highly sensitive, the specificity of FDG remains suboptimal for differentiating malignant from benign nodules, particularly in areas where fungal lung diseases are prevalent. Thus, a cancer-specific imaging probe is greatly needed. In this study, we tested the hypothesis that a PET radiotracer (S)-4-(3-[^18^F]-fluoropropyl)-L-glutamic acid (FSPG) improves the diagnostic accuracy of IPNs compared to ^18^F-FDG PET/CT.

**Methods:**

This study was conducted at a major academic medical center and an affiliated VA medical center. Twenty-six patients with newly discovered IPNs 7-30mm diameter or newly diagnosed lung cancer completed serial PET/CT scans utilizing ^18^F-FDG and ^18^F-FSPG, without intervening treatment of the lesion. The scans were independently reviewed by two dual-trained diagnostic radiology and nuclear medicine physicians. Characteristics evaluated included quantitative SUVmax values of the pulmonary nodules and metastases.

**Results:**

A total of 17 out of 26 patients had cancer and 9 had benign lesions. ^18^F-FSPG was negative in 6 of 9 benign lesions compared to 7 of 9 with ^18^F-FDG. ^18^F-FSPG and ^18^F-FDG were positive in 14 of 17 and 12 of 17 malignant lesions, respectively. ^18^F-FSPG detected brain and intracardiac metastases missed by ^18^F-FDG PET in one case, while ^18^F-FDG detected a metastasis to the kidney missed by ^18^F-FSPG.

**Conclusion:**

In this pilot study, there was no significant difference in overall diagnostic accuracy between ^18^F-FSPG and ^18^F-FDG for the evaluation of IPNs and staging of lung cancer. Additional studies will be needed to determine the clinical utility of this tracer in the management of IPNs and lung cancer.

## Introduction

Lung cancer remains the leading cause of cancer-related death in the United States. The overall 5-year survival from lung cancer remains poor at approximately 20% [1]. Early detection is key to survival as mortality increases with advanced stage [2]. One of the factors that contributes to this high mortality is the delay in definitive diagnosis when an indeterminate pulmonary nodule (IPN) is newly discovered. However, even in high-risk patients, most IPNs are benign [3, 4]. Based on risk assessment, one management option for a newly diagnosed intermediate-risk lung nodule is to repeat imaging over time with the risk of delaying treatment and missing a potential chance of cure. The second option is to proceed with tissue diagnosis at the time of discovery, but this strategy leads to unnecessary healthcare spending, invasive procedures, anxiety, and morbidity [2, 5, 6].

^18^F-FDG PET/CT is a functional imaging modality recommended for the evaluation of intermediate-risk IPNs [7–9], although this practice is controversial [10]. While highly sensitive, the specificity of ^18^F-FDG remains variable for differentiating malignant from benign lesions, at approximately 40–60%, particularly in areas with endemic fungal lung disease [10–12]. Furthermore, the sensitivity and specificity of ^18^F-FDG to diagnose suspicious IPNs vary across clinical context, histologic subtype, and nodule size [13–15]. Several other nuclear imaging probes have been tested to differentiate benign vs malignant pulmonary nodules, but none have been validated to show superiority to ^18^F-FDG PET/CT [16–21]. Thus, there is an unmet need for a cancer-specific imaging probe to better differentiate benign from malignant pulmonary nodules.

(*S*)-4-(3-[18F]-fluoropropyl)-L-glutamic acid (^18^F-FSPG) is an ^18^F-labeled glutamic acid derivative designed to target tumor-specific adaptations of intermediary metabolism. ^18^F-FSPG is taken up via the xCT transporter, a glutamate-cystine exchanger (SLC7A11/SLC3A2 (CD44) heterodimer) that transports L-cystine (Cys-S-S-Cys) into the cell and L-glutamate to the extracellular compartment [22]. ^18^F-FSPG has shown promise as a cancer-imaging probe in a variety of preclinical and clinical settings [22–29]. ^18^F-FSPG has been evaluated in patients with several tumors, including breast, lung, prostate, colorectal, non-Hodgkin lymphoma, head and neck, hepatocellular carcinoma and brain lesions with promising results [24–29]. ^18^F-FSPG may also have superior accuracy in correctly identifying benign lesions as compared to ^18^F-FDG [23, 30].

Given these encouraging clinical and preclinical data, we investigated the hypothesis that in patients with IPNs, PET/CT imaging with ^18^F-FSPG improves diagnostic accuracy compared to ^18^ F-FDG PET/CT. In patients with newly diagnosed, untreated lung cancer, we also compared their accuracy for staging and correlated ^18^F FSPG uptake with xCT and CD44 expression.

## Methods

### Study design

This was a pilot, open-label, prospective non-randomized clinical study. Our primary endpoint was the accuracy of ^18^F FSPG and ^18^F FDG PET/CT in discriminating between benign and malignant lesions in patients with newly discovered IPNs. Our secondary endpoint was the accuracy of ^18^F FSPG and ^18^F FDG PET/CT for initial staging in patients with newly diagnosed treatment-naïve lung cancer. We also investigated the correlation of ^18^F FSPG uptake with CD44 and xCT transporter expression in malignant lesions. This study was approved by the Internal Review Boards at Vanderbilt University Medical Center (IRB# 150392) and of the Nashville VA Medical Center (IRB# 1205000) and registered in Clinicaltrials.gov (NCT02448225). Informed consent was obtained from all patients before enrollment.

### Patients selection (inclusion/exclusion criteria)

Vanderbilt University Medical Center’s (VUMC) and Nashville VA Medical Center’s (VAMC) Institutional Review Boards (IRBs) approved this study under the purview of the US Food and Drug Administration investigational new drug (IND) application 124202 ([^18^F]FSPG). Prospective participants from VUMC and VAMC were recruited by the study team members. Inclusion criteria included adult patients, age 40 to 80, with a newly detected IPN (7-30mm in diameter) that had not previously been evaluated, or newly diagnosed, untreated primary lung cancer 7 mm or more in diameter, and who had an indication for ^18^FDG PET/CT as part of standard of care evaluation. Exclusion criteria included known active lung infection, pregnant or lactating patients, body weight greater than 400 pounds, a body habitus or disability that would not permit the imaging protocol to be performed, previous radiation or systemic treatment for cancer of any type within 1 year, and, if no tissue diagnosis, expected lifespan less than 2 years, which would preclude appropriate follow up.

### PET/CT protocol

All patients underwent a standard of care whole body ^18^F-FDG PET/CT scan for the evaluation of an IPN or lung mass. A similar protocol was followed for obtaining the ^18^F-FSPG PET/CT images. ^18^F-FSPG scans were performed within 30 days of the ^18^F-FDG scans with no intervening treatment of the lesion, except for 2 patients who had 34 and 63 days in between scans but no intervening treatment. The patients were asked to have nothing to eat or drink (NPO) after midnight on the day of the scans. Fasting blood glucose levels were measured prior to initiation of the exam to ensure that the levels were below 200 mg/dL. Standard of care scan from the vertex to mid-thigh was performed approximately 60 minutes after the IV administration of ^18^F-FDG (dose range 10.1 to 24.5 mCi) or ^18^F-FSPG (dose range 7.5 to 9 mCi) with the patient in the supine position. Images were acquired either on a GE Discovery STE (VUMC) or GE Discovery VCT (VAMC) PET/CT scanner with emission imaging in 3D mode with scatter correction. Low dose CT scans were obtained for attenuation correction and anatomic localization.

### Imaging analysis

^18^F-FDG and ^18^F-FSPG PET/CT studies were reviewed independently by two dual-trained diagnostic radiology and nuclear medicine physicians blinded to each other’s interpretation. A third radiologist was available for ties, but this was not necessary. Maximum standardized uptake values (SUV_max_) were normalized to lean body mass and measured with a 1 cm diameter round region of interest over the area of greatest uptake in the lesion being measured. Regions of interest were drawn around the IPNs as well as any suspicious areas of metastasis. SUV_max_ values from the IPNs, metastases and blood pool in the ascending aorta were recorded. These measurements were performed by using Xeleris 4.0 workstation hardware and software from GE Healthcare, Waukesha, MI, USA.

### Radiotracer production

^18^F-FSPG was produced by the Vanderbilt Center for Molecular Probes Radiochemistry Core Laboratory in accordance with the Chemistry, Manufacturing, and Control (CMC) sections of the IND referenced above. Tracers met all USP <823> requirements for sterile, injectable PET radiopharmaceuticals. The tracers used for this PET study were previously evaluated in humans at VUMC and other sites with no adverse side effects, such as anaphylactic reactions, allergic reactions, any morbidity, or mortality observed. ^18^F-FSPG is obtained by radiolabeling of the protected precursor di-*tert*butyl (2*S*,4*S*)-2-(3-((naphthalen-2-ylsulfonyl)oxy)propyl)-4-(tritylamino)pentanedioate) (PI-021) with [18F]fluoride. After acidic deprotection, the tracer is purified over cartridges and finally formulated for intravenous injection by passing the solution through a 0.22-μm sterile filter. The synthesis is performed within an automated synthesis module in a lead-shielded hot cell.

### Immunohistochemistry of xCT transporter and CD44 expression

Tumor tissue from biopsies or surgical excisions performed as standard of care for pathologic examinations were obtained before or after PET/CT imaging for immunohistochemistry (IHC) studies for xCT and CD44 expression levels. Tissue processing and IHC analysis of formalin-fixed, paraffin-embedded tissue sections were performed by VUMC Translational Pathology Shared Resource and as detailed in previous publications [31, 32]. Briefly, 5-um thick whole tissue sections were transferred onto poly-L-lysine-coated adhesive slides and dried at 60°C for 60 minutes. Slides were then placed on the Leica Bond-Rx IHC stainer. All steps besides dehydration, clearing and coverslipping were performed on the Leica Bond-RX. Slides were deparaffinized. Heat induced antigen retrieval was performed on the Bond-Rx using their Epitope Retrieval 1 solution for 20 minutes (anti-CD44) and Epitope Retrieval 2 solution for 20 minutes (anti-xCT). Slides were incubated with anti-CD44 (cat# 3570S, Cell Signaling Technology, Danvers, MA) for one hour at a dilution of 1:500 and with anti-xCT (cat# 12691S, Cell Signaling Technology, Danvers, MA) for one hour at a dilution of 1:100. The Leica Bond Refine (Polymer) detection system was used for visualization. Slides were then dehydrated, cleared and coverslipped. The level of expression of xCT and CD44 proteins in the cytoplasmic membrane of tumor cells were examined by an experienced pathologist who was blinded to any patient and imaging information. The percentage of tumor cells positive for the marker and the intensity of staining were evaluated, the latter using a scale of 0 (none), 1+ (weak), 2+ (intermediate), and 3+ (strong) with a sample being reported as positive if greater than 10% of the tumor cells in the sample were positively stained with any intensity. The correlation between the intensity of IHC staining and SUV_max_ of the corresponding lesion on the PET/CT was assessed using Spearman correlation.

### Statistical analysis

Descriptive statistics, reported as means and standard deviations or median and interquartile range for continuous variables and percentages and frequencies for categorical parameters, were calculated. Sensitivity, specificity, positive predictive value (PPV), negative predictive value (NPV), accuracy, and receiver operating characteristic (ROC) curves were generated for ^18^F-FDG and ^18^F-FSPG. McNemar test was used to compare sensitivity, specificity, and accuracy. Bootstrap method was used to compare the AUCs of the two ROC curves. Spearman correlation was used to assess the association between immunohistochemical staining (CD44 and xCT expression levels) and the SUV_max_ of ^18^F-FDG and ^18^F-FSPG tests. Statistical analysis was conducted using R version 3.6.1.

## Results

This study was terminated before the target 30-patient goal due to the SARS CoV 2 pandemic and difficulty with subject enrollment and funding. Forty-six patients were enrolled in the study, of which 27 completed both ^18^F-FSPG and ^18^F-FDG PET scans. One patient was excluded from the analysis because of treatment of the nodule of interest in between scans. Baseline characteristics of the participants are presented in **Table 1**. Twenty-six subjects underwent ^18^F-FSPG and ^18^F-FDG PET scans with a median time between the scans of 17 days (IQR, 12–21). The median age at the time of the scans was 65 years old, and 69% of the subjects were males. Most patients (24/26) were either current or former smokers. The median pack-year-history was 50 (IQR, 45–60) in those diagnosed with malignancy compared to 40 (IQR, 0–50) in those with benign lesions. A total of 17 patients were diagnosed with cancer while 9 patients had benign lesions. Of these, 5 patients had no evidence of malignancy on biopsy, and 4 patients had nodule stability or regression after 2 years of follow up. All IPNs in this cohort were solid, except for one ground glass nodule that was diagnosed as adenocarcinoma. The median lesion size was similar in benign lesions (1.6 cm) and malignant lesions (1.5 cm), and 81% of them were located in the upper lobes.

**Table 1:** Baseline characteristics.

Characteristics	Benign (N = 9)	Malignant (N = 17)
Age—years, Median (IQR)	62 (54–68)	66 (58–70)
Male sex–no. (%)	6 (67)	12 (71)
Smoking History		
Current–no. (%)	5 (56)	6 (35)
Former–no. (%)	2 (22)	11 (65)
Never–no. (%)	2 (22)	0 (0)
Pack Year, Median (IQR)	40 (0–50)	50 (45–60)
BMI, Median (IQR)	33 (22–41)	28 (23–34)
COPD–no. (%)	3 (33)	10 (59)
Days Between Scans, Median (IQR)	19 (14–20)	15 (11–23)
Lesion Size (cm), Median (IQR)	1.6 (0.9–1.8)	1.5 (1.2–3.8)
Spiculation–no. (%)	2 (22)	7 (41)
Nodule Location		
Lower lobe–no. (%)	2 (22)	4 (24)
Upper lobe–no. (%)	7 (78)	13 (76)
Histologic Diagnosis		
NSCLC–no.		12
Malignant (Unknown type)–no.		2
Renal cell carcinoma–no.		1
Sarcomatoid carcinoma–no.		1
SCLC–no.		1
Stage		
IA–no.		5
IIB–no.		1
III–no.		5
IV–no.		3
Stage IV (SCLC)–no.		1
Metastatic from RCC–no.		1
Unknown–no.		1

BMI-Body Mass Index

COPD-Chronic Obstructive Pulmonary Disease

NSCLC-Non-Small Cell Lung Cancer

SCLC-Small Cell Lung Cancer

RCC-Renal Cell Carcinoma

Of the malignant lesions, 12 were NSCLC. The other histologic diagnoses included 1 recurrent metastatic renal cell carcinoma, 1 sarcomatoid carcinoma, 1 small cell carcinoma, and 2 unknowns. One of the unknowns was empirically radiated for presumed malignancy given the nodule characteristics and very high-risk patient. The other unknown was also empirically radiated after nodule growth over time and biopsy with highly atypical cells. Of the patients with confirmed NSCLC, 8 had advanced stage (III and IV) while 4 patients had Stage I or II disease.

**Fig 1:**
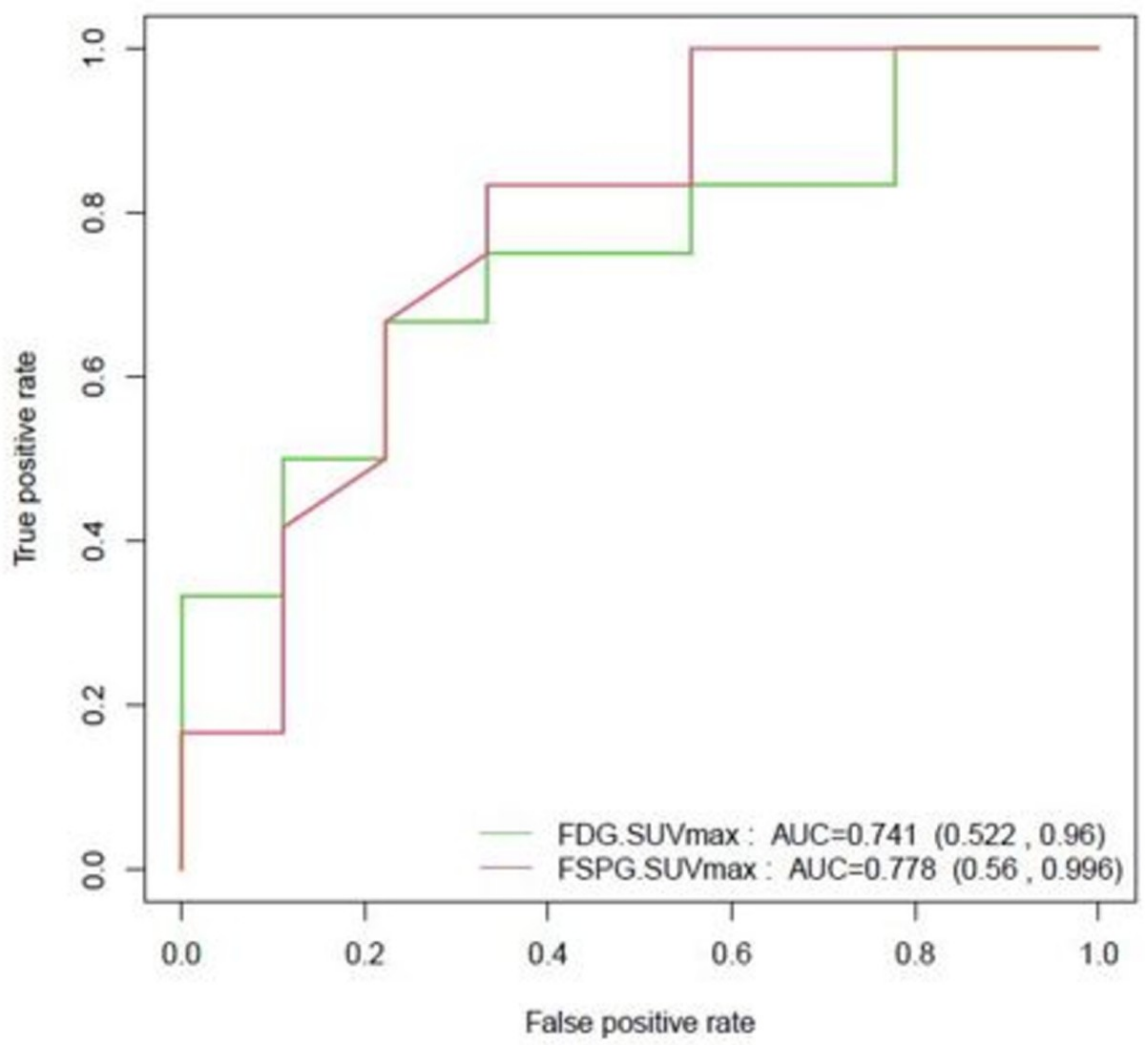
^18^F-FSPG and ^18^F-FDG ROC curves for IPNs (N = 21).

^18^F-FSPG and ^18^F-FDG PET/CT were positive in 14 of 17 and 12 of 17 malignant lesions respectively. When subdivided into IPNs (7-30mm) and lung masses (>30mm), ^18^F-FSPG and ^18^F-FDG PET/CT were positive in 9 of 12 and 7 of 12 malignant IPNs respectively, and 5 of 5 lung masses. The two nodules that were negative with ^18^F-FDG but positive on ^18^F-FSPG were solid. One nodule measured 1.7cm and was diagnosed as Stage IIB NSCLC. The other nodule measured 1.2 cm and was presumed to be malignant (interval growth and highly atypical cells on biopsy) and empirically radiated. ^18^F-FSPG was negative in 6 of 9 benign lesions compared to 7 of 9 with ^18^F-FDG. The sensitivity of ^18^F-FSPG and ^18^F-FDG for IPNs was 75% and 58% respectively, the specificity was 67% and 78% respectively, and the accuracy was 71% and 67% respectively. Sensitivity, positive predictive value (PPV) and accuracy slightly improved for both tracers after adding lung masses to the analysis. The median SUVmax was lower in benign lesions for both ^18^F-FSPG and ^18^F-FDG. ^18^F-FSPG SUVmax was lower in both benign and malignant lesions compared to ^18^F-FDG. These results are presented in **Tables 2 and 3**. The ROC curves for ^18^F-FSPG and ^18^F-FDG are shown in **Fig 1**. Overall, there was no significant difference between the two AUCs (p = 0.64).

**Table 2:** ^18^F-FSPG and ^18^F-FDG evaluation of primary lesion.

	IPNs (7-30mm)		Mass (>30mm)
	Malignant (N = 12)	Benign (N = 9)	Malignant (N = 5)
^**18**^ **F-FDG**			
Positive–no. (%)	7 (58)	2 (22)	5 (100)
Negative–no. (%)	5 (42)	7 (78)	0
SUVmax, Median (IQR)	2.2 (1.6–4.3)	1.4 (0.7–1.8)	11.3 (10.1–17.1)
^**18**^ **F-FSPG**			
Positive–no. (%)	9 (75)	3 (33)	5 (100)
Negative–no. (%)	3 (25)	6 (67)	0
SUVmax, Median (IQR)	1.2 (1–1.7)	0.7 (0.4–1.0)	8.5 (6.7–8.8)

SUVmax–Maximum standardized uptake value

^18^F-FSPG SUVmax >1 and ^18^F-FDG SUVmax >2 defines a positive result

**Table 3:** Performance Comparison of ^18^F-FSPG and ^18^F-FDG for IPNs and combined IPNs and lung masses.

	IPNs (7-30mm) N = 21		All Pulmonary Lesions N = 26	
	^18^F-FDG (95% CI)	^18^F-FSPG (95% CI)	^18^F-FDG (95% CI)	^18^F-FSPG (95% CI)
Sens (%)	58 (0.30–0.86)	75 (0.51–1.00)	71 (0.49–0.92)	82 (0.64–1.00)
Spec (%)	78 (0.51–1.00)	67 (0.36–0.98)	78 (0.51–1.00)	67 (0.36–0.98)
PPV (%)	78 (0.51–1.00)	75 (0.51–1.00)	86 (0.67–1.00)	82 (0.64–1.00)
NPP (%)	58 (0.30–0.86)	67 (0.36–0.98)	58 (0.30–0.86)	67 (0.36–0.98)
Accuracy (%)	67 (0.47–0.87)	71 (0.52–0.91)	73 (0.56–0.90)	77 (0.61–0.93)

^18^F-FSPG and ^18^F-FDG PET/CT were positive in all patients with metastatic disease except for two patients. One patient had stage II NSCLC cancer who had positive LN involvement on biopsy but negative PET scans. Notably, this was one of the subjects who had a false negative ^18^F-FDG PET. The other patient had multiple ground-glass nodules that were falsely negative with both tracers and ultimately diagnosed with stage III adenocarcinoma on biopsy. ^18^F-FSPG scans were positive in all sites where ^18^F-FDG PET/CT showed signs of metastasis except in 2 patients. ^18^F-FSPG detected brain and intracardiac metastases not visualized by ^18^F-FDG PET/CT in one case [33] while a biopsy proven metastatic lesion to the kidney was not seen on the ^18^F-FSPG PET/CT but seen on ^18^F-FDG PET/CT.

**Fig 2:**
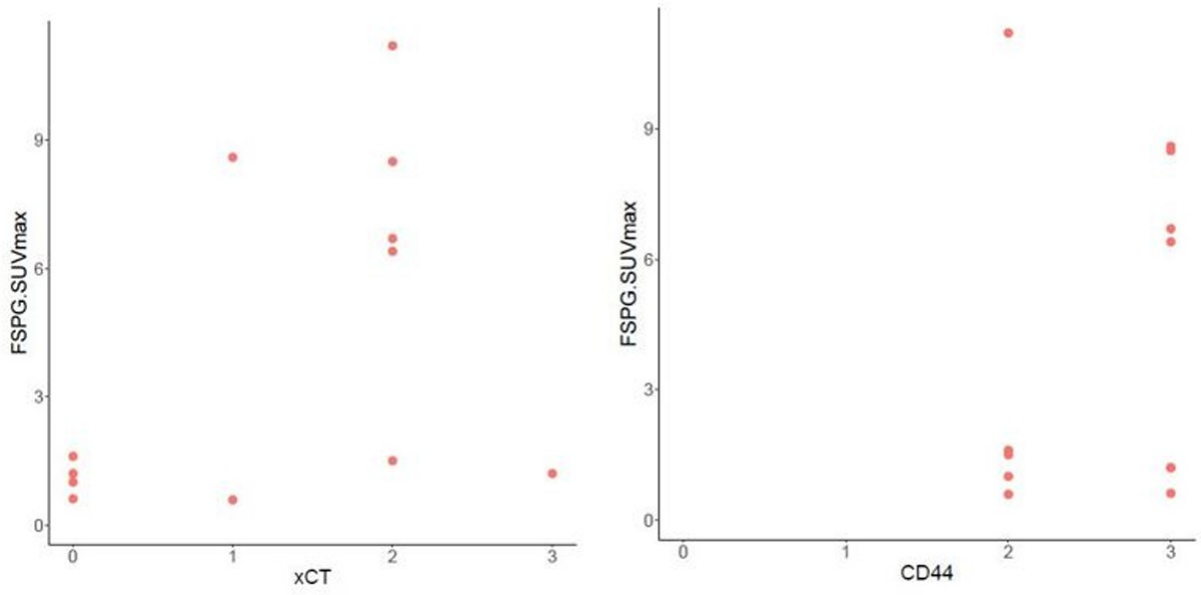
^18^F-FSPG SUVmax correlation with xCT (spearman correlation 0.440, p = 0.153) and CD44 (spearman correlation 0.172, p = 0.594) in cancer cases.

Pathology slides for IHC analysis were available on 12 of 17 patients with cancer, including the renal cell cancer case. We found no correlation between ^18^F FSPG SUVmax and the intensity of staining of xCT (Spearman correlation 0.440, p = 0.153) and between ^18^F FSPG SUVmax and the intensity of staining of CD44 (Spearman correlation 0.172, p = 0.594) (**Fig 2**). Of note, CD44 intensity was moderate or high (+2 or +3) in all our samples.

**Fig 3:**
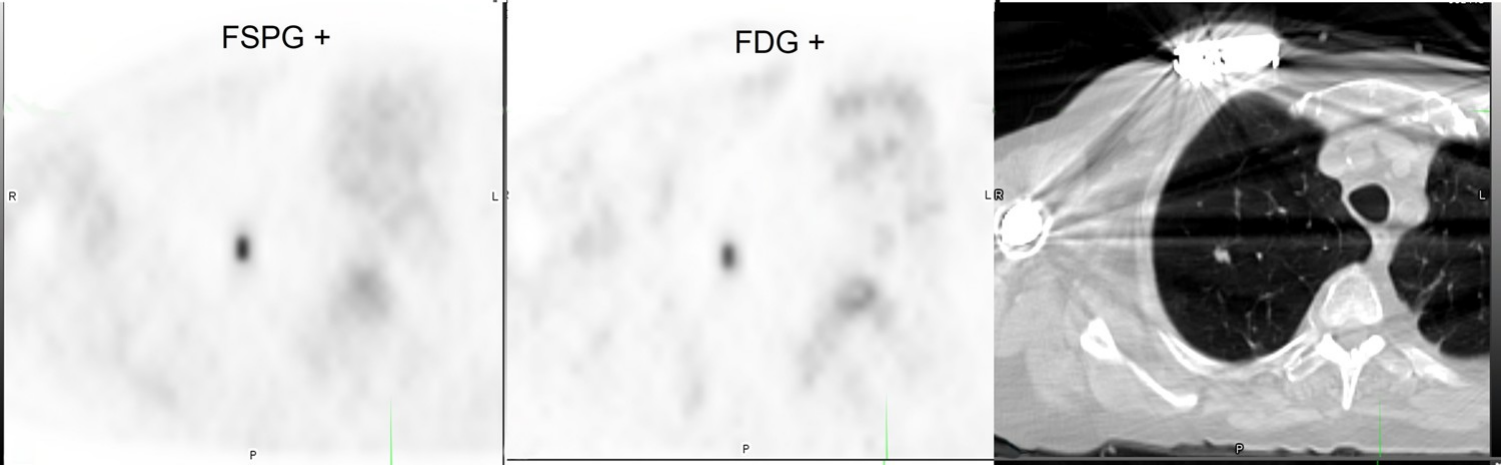
Subject with positive ^18^F-FSPG (SUVmax 3.5) and ^18^F-FDG (SUVmax 7.4) PET/CT.

**Fig 4:**
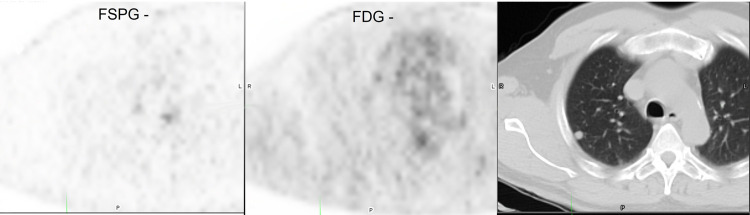
Subject with negative ^18^F-FSPG (SUVmax 0.4) and ^18^F-FDG (SUVmax 0.54) PET/CT.

**Fig 5:**
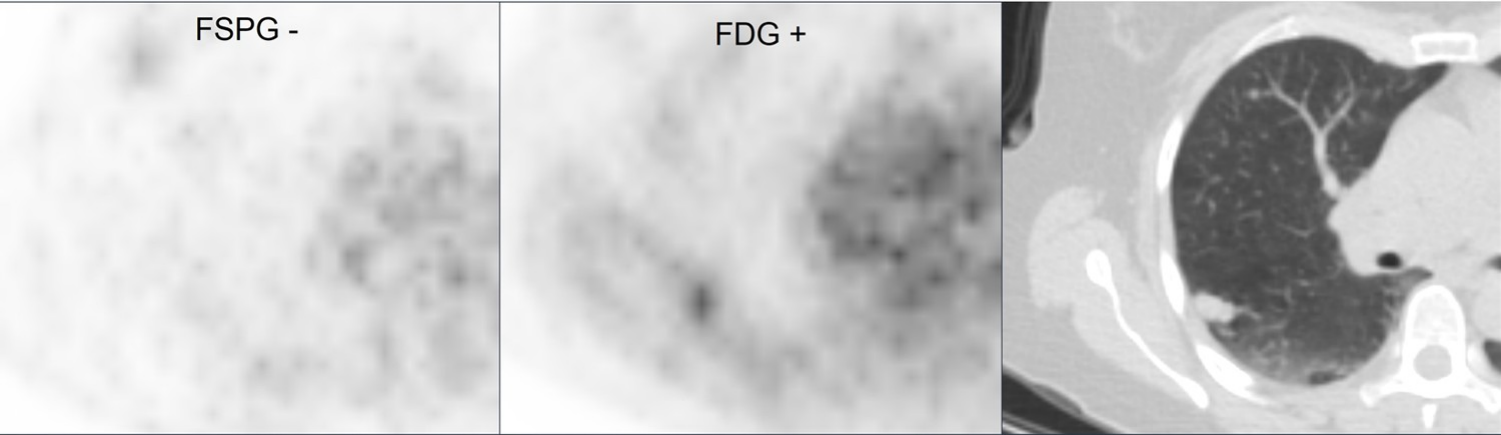
Subject with negative ^18^F-FSPG (SUVmax 0.9) and positive ^18^F-FDG (SUVmax 2.4) PET/CT.

**Fig 6:**
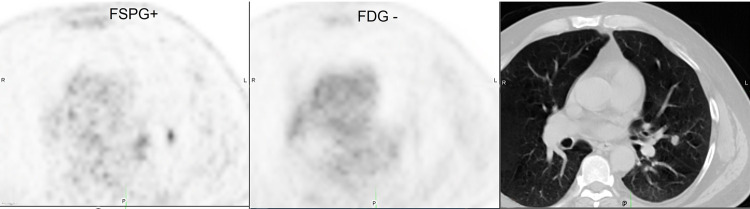
Subject with positive ^18^F-FSPG (SUVmax 2.02) and negative ^18^F-FDG (SUVmax 0.7) PET/CT.

Representative images from 4 subjects showing positive and negative ^18^F-FSPG and ^18^F-FDG PET/CT scans are shown in **Figs 3**–**6**.

## Discussion

In this prospective pilot study, we compared the accuracy of ^18^F-FSPG PET/CT to ^18^F-FDG PET/CT in discriminating between benign and malignant lesions in patients with newly discovered IPNs. We found that both tracers had similar accuracy in our small cohort; however, we were underpowered to detect a significant difference between these two tracers. In patients diagnosed with lung cancer, we also found no significant difference between the two tracers for initial lung cancer staging.

To our knowledge, this is the first prospective and largest study of ^18^F-FSPG PET/CT for the evaluation of IPNs and staging of lung cancer. Unlike prior studies, we enrolled patients with newly discovered IPNs, which allowed us to compare the performance of ^18^F-FDG and ^18^F-FSPG prospectively. ^18^F-FSPG detected more malignant IPNs (14 of 17 cancers) compared to ^18^F-FDG (12 of 17 cancers). If we exclude the RCC case that was missed by both tracers, ^18^F-FSPG detected 14 of 16 lung cancers and ^18^F-FDG 12 of 16 lung cancers. The sensitivity observed with ^18^F-FDG (71% overall) is partly explained by the histology of the cases missed and the nodule size, both known to decrease the sensitivity of ^18^FDG PET [14]. Beak and colleagues evaluated ^18^F-FSPG in 10 patients with known NSCLC and a positive ^18^F-FDG PET/CT and found that all patients had positive ^18^F-FSPG scans [25]. Our results differ from this study as we had a few false negatives; however, the patients in Beak, et al, had positive ^18^F-FDG PET/CT, were known to have lung cancer and had a larger average lesion size compared to our study, which included patients with undiagnosed IPNs >7mm.

^18^F-FSPG appears to have the potential to correctly identify benign lesions [23, 30] and thus potentially better specificity compared to ^18^F-FDG. In this study, both tracers appear to have similar specificity, although we only had 9 benign nodules and were underpowered to detect a difference. While the overall SUV_max_ was lower with ^18^F-FSPG, this did not result in better discrimination between benign and malignant lesions. Of the 3 patients in our cohort with benign lesions that had positive ^18^F-FSPG scans, 2 underwent bronchoscopies to obtain biopsies and one was followed with interval imaging. Although biopsies did not show cancer, no firm diagnosis was secured in either case. Histoplasma is an endemic fungus in the Mississippi and Ohio River Valleys where our institutions are located, and a common cause of false positive results on ^18^F-FDG PET [12]. It is possible that some of these false positives could represent granulomatous inflammation secondary to Histoplasma akin to what is seen in sarcoidosis, which has shown to have high ^18^F-FSPG uptake [30].

One potential strength of ^18^F-FSPG appears to be its ability to detect metastatic foci from lung cancer in tissues with high physiologic ^18^F-FDG uptake such as the brain, which is a major limitation of ^18^F-FDG. In a small pilot study that included patients with NSCLC metastatic to the brain, all 3 patients with metastatic brain lesions from NSCLC had a positive ^18^F-FSPG scan [27]. In our study, we had a patient with cardiac and cerebellar metastases that were not evident with ^18^F-FDG but easily identified with ^18^F-FSPG [33]. While these results are encouraging, additional studies involving patients with metastatic disease to the brain will be needed to confirm these early findings. Although the kidneys are not a common metastatic site for NSCLC [34], we had one patient in our cohort with an intensely ^18^F-FDG avid right renal metastasis that was not seen in the ^18^F-FSPG scan.

^18^F-FSPG is taken up via the xCT transporter, a glutamate-cystine exchanger (SLC7A11/SLC3A2 (CD44) heterodimer) that transports L-cystine (Cys-S-S-Cys) into the cell and L-glutamate to the extracellular compartment [22]. Overexpression of xCT provides tumor cells a survival advantage as this allows them to maintain high levels of glutathione synthase to detoxify reactive oxygen species efficiently [22]. In a pilot study by Beak and colleagues that included 10 patients with ^18^F-FDG PET positive NSCLC and 5 patients with breast cancer, immunohistochemical (IHC) analyses on these subjects’ pathology samples showed significant correlation between ^18^F-FSPG uptake and protein expression of both the SLC7A11 subunit of system xC- and the stem cell marker CD44. In breast tumor samples specifically, IHC showed that absence of CD44 correlated with low signal from ^18^F-FSPG-PET, even if the SLC7A11 subunit was present, indicating possible importance of CD44 co-expression for system xC- function [25]. In our study, we found no correlation between the intensity of staining of xCT and CD44 with ^18^F-FSPG SUV_max_. Even though no statistically significant correlation was found between xCT intensity and FSPG SUV_max_, most cases with low xCT IHC score had a relatively low FSPG SUV_max_ while most cases with moderate or high xCT IHC score had a relatively high ^18^F-FSPG SUV_max_ (**Fig 2**). The lack of significant correlation might be explained by the small sample size. CD44 IHC score was high (+2 or +3) in all of our cases. One possible explanation, as previously noted, is that CD44 co-expression is necessary for the system to properly function [25], but it seems that high CD44 does not lead to high FSPG uptake if the xCT subunit is not present or is in low concentrations. A lab error could also account for the high CD44 IHC score, although we followed the same procedures described in our previously published studies [31, 32]. Given we do not have low CD44 IHC score, we could not assess whether low or absent CD44 could be associated with lower FSPG uptake regardless of xCT expression as seen in other studies [25].

Besides lung cancer, ^18^F-FSPG has been evaluated in patients with several other tumors including breast, prostate, colorectal, non-Hodgkin lymphoma, head and neck, hepatocellular carcinoma, and brain lesions [24–29]. Like these studies, our study highlights the promising clinical potential of ^18^F-FSPG, but the similar accuracies found in our study should be interpreted as hypothesis generating given our small sample and limited statistical power to detect a difference.

Our study has several limitations. First, the study was terminated before achieving our 30-patient goal due to the SARS CoV2 pandemic and difficulty with enrollment. Only 9 subjects were diagnosed with benign lesions in this cohort, and to show superior specificity, a larger number of benign nodules was needed. Additionally, we have no formal diagnosis for patients with benign lesions, and pathology slides for IHC were available in 12 of 17 patients diagnosed with malignancy and none in those diagnosed with benign disease. Time between scans could potentially bias the results toward correct diagnosis with ^18^F-FSPG as malignant nodules might grow during this time interval and benign nodules might decrease in size. There was a total of 6 subjects that underwent ^18^F-FSPG scans more than 3 weeks after the ^18^F-FDG scans. One subject had a benign nodule with negative ^18^F-FSPG and ^18^F-FDG scans. This nodule did not significantly change during this time interval. The other 5 subjects had cancer and were all positive with both tracers. Only two subjects had their scans more than 1 month apart: one with stage IV sarcomatoid carcinoma (34 days apart) and one with stage IA adenocarcinoma (63 days apart). Although the nodule diagnosed as sarcomatoid carcinoma grew from 1.7cm to 2cm, we believe this likely did not affect the results given the high avidity of the nodule. The other nodule did not significantly change in the size over this time interval. Our investigation used identical PET/CT imaging protocols for both ^18^F-FSPG and ^18^F-FDG. Further work, especially in animal models, might demonstrate superior performance of ^18^F-FSPG if the imaging protocol were maximized for this radiopharmaceutical, such as using earlier or later imaging after injection, and/or imaging of dynamic uptake and/or washout. Thus, with proper maximization of the imaging protocol and larger sample size, ^18^F-FSPG might show superior accuracy to ^18^F-FDG for PET/CT evaluations of IPNs.

## Conclusion

In this pilot study, there was no significant difference in overall diagnostic accuracy between ^18^F-FSPG and ^18^F-FDG for the evaluation of IPNs and staging of lung cancer, but we were limited by the small sample size. Additional studies will be needed to determine the clinical utility of this tracer in the management of IPNs and lung cancer.

## Supporting information

S1 Checklist TREND checklist. (PDF)

S1 Fig Modified CONSORT diagram. (DOC)

S1 File Study protocol. (DOCX)
