# Supplementary material for: 18F-FSPG PET imaging for the evaluation of indeterminate pulmonary nodules
Source: PLoS One. 2022 Mar 16;17(3):e0265427. doi: 10.1371/journal.pone.0265427 (PMC8926263; doi:10.1371/journal.pone.0265427)
Supplement: S1 Fig — (DOC) [file pone.0265427.s002.doc]

**CONSORT Flow Diagram**

Enrolled (n=46)

Excluded (n= 19)

- Did not undergo PET with ^18^F-FSPG (n=18)

- Withdrew consent (n=1)

Completed the study and included in the final analysis (n=26)

Underwent PET with ^18^F-FSPG and ^18^F-FDG (n=27)

Excluded (n= 1)

- Received treatment of the lesion of interest between scans
