# Supplementary material for: 18F-FSPG PET imaging for the evaluation of indeterminate pulmonary nodules
Source: PLoS One. 2022 Mar 16;17(3):e0265427. doi: 10.1371/journal.pone.0265427 (PMC8926263; doi:10.1371/journal.pone.0265427)
Supplement: S1 File — (DOCX) [file pone.0265427.s003.docx]

**THO 1524: PET Imaging of Lung Cancer and Indeterminate Pulmonary Nodules with ^18^F-FSPG**

**Ver: 02DEC2021**

**Sponsor- Investigator**

Chirayu Shah, MD

Associate Professor of Clinical Radiology and Radiological Sciences

Vanderbilt University Medical Center

Nashville, TN

**Statistician**

Heidi Chen, PhD

Associate in Biostatistics, Division of Cancer Biostatistics

Vanderbilt-Ingram Cancer Center

Vanderbilt University Medical Center, Nashville, TN

**Additional Site**

Tennessee Valley Health System Nashville

Site Principal Investigator Chirayu Shah, MD

Table of Contents

[1. Background 3](#_Toc23328097)

[2. Objectives, Rationale, and Specific Aims 8](#_Toc23328098)

[3. Animal Studies and Previous Human Experience 9](#_Toc23328099)

[4. Patient Eligibility & Inclusion / Exclusion Criteria 10](#_Toc23328100)

[A. Inclusion Criteria 10](#_Toc23328101)

[B. Exclusion Criteria 10](#_Toc23328102)

[5. Enrollment/Randomization 11](#_Toc23328103)

[Patient Registration 12](#_Toc23328104)

[6. Data Management and Monitoring 12](#_Toc23328105)

[7. Study Procedures & Data Collection 13](#_Toc23328106)

[Baseline Imaging: CT 13](#_Toc23328107)

[Imaging Protocol: ^18^F-FSPG (Figure 1) 13](#_Toc23328108)

[Radiotracer Production 15](#_Toc23328109)

[^18^F-FSPG PET Procedure and Dynamic Data Analysis 15](#_Toc23328110)

[Clinical Data Analysis 15](#_Toc23328111)

[Rationale for Size Criteria 16](#_Toc23328112)

[8. Risks 18](#_Toc23328113)

[Radiopharmaceutical 18](#_Toc23328114)

[Injection 18](#_Toc23328115)

[Reproductive Risk 18](#_Toc23328116)

[Dose Rationale 18](#_Toc23328117)

[Radiation Safety 18](#_Toc23328118)

[Toxicity Studies 19](#_Toc23328119)

[9. Reporting of Adverse Events or Unanticipated Risks to Participants or Others 19](#_Toc23328120)

[Serious Adverse Events (SAE) are defined in accordance with the NCI’s CTCAE Ver. 4.0. 20](#_Toc23328121)

[10. Study Withdrawal / Discontinuation 21](#_Toc23328122)

[11. Statistical Considerations 21](#_Toc23328123)

[Study Design 21](#_Toc23328124)

[Sample Size and Study Power 22](#_Toc23328125)

[Data Analysis 22](#_Toc23328126)

[12. Privacy / Confidentiality 23](#_Toc23328127)

[13. Follow-up and Record Retention 23](#_Toc23328128)

[Study Procedure Calendar (2-year follow-up by CT only needed for subjects without a definitive diagnosis of the nodule or mass by biopsy) 24](#_Toc23328129)

[14. References 25](#_Toc23328130)

**1. Background**

**Lung Cancer and Indeterminate Pulmonary Nodules (IPNs)**: The estimated number of patients with newly diagnosed lung cancer in the US for 2013 is 228,190 (118,080 men and 110,110 women), with an estimated 159,480 deaths (87,260 men, 72,220 women). Lung cancer now is, and will be for years to come, the leading cause of cancer death in both the US and the world. The five-year relative cancer survival of all stages and all types combined (2002-2008) ranges from 17% for lung cancer to nearly 100% for prostate cancer ([1](#_ENREF_1)). The future U.S. cancer burden, estimated from the current and projected size and age demographics of the U.S. population, is expected to more than double from 1.4 million in 2000 to almost 3.0 million in 2050. The lifetime risk of developing cancer of the lung/bronchus is 1 in 12 for men and 1 in 16 for women ([2](#_ENREF_2)). The primary risk factor for lung cancer is smoking, with the percentage of the southeastern US population estimated to be smokers significantly higher than for the general US population, with correspondingly higher incidence and death rates from all tobacco-related cancers.

Two contributors to the high mortality of lung cancer are the frequent advanced stage of the disease when discovered and the delay in diagnosis of cancer in a newly discovered IPN. This delay in definitive diagnosis of an IPN can potentially allow time for metastasis to occur during the follow-up interval. The exciting results of the National Lung Screening Trial (NLST) demonstrated an approximately 20% reduction in lung cancer specific mortality *via* low-dose computed tomography (LDCT) screening of high-risk patients. As a result, the US Preventive Services Task Force has recommended annual LDCT screening for an estimated 7.4 million Americans at high risk for lung cancer ([3](#_ENREF_3)). However, even in high-risk patients, most IPNs were benign. Specifically, 24.2% of LDCT screening scans had IPNs requiring further evaluation (usually serial CTs for up to 2 years), with 96.4% of these positive screening CTs eventually found to be false positive ([4](#_ENREF_4)). Furthermore, 8.3% of all positive scans had a subsequent ^18^F-FDG PET/CT scan as part of the diagnostic process. Since IPNs in high risk patients must be followed with serial LDCT exams ([5](#_ENREF_5)), and guidelines suggest diagnosis with ^18^F-FDG-PET/CT scans for lesions larger than 8 mm that have a greater than a 5% likelihood of cancer ([6](#_ENREF_6)), patients may receive up to 5 additional LDCT scans, and possibly an ^18^F-FDG PET/CT scan, to follow an IPN. These subsequent scans are at considerable time/expense to both the patient and the healthcare system, when over 96% of these nodules will be benign. The economic burden of this disease is staggering. The annual cost of >150,000 deaths/y in the US from lung cancer is >$12 billion ([7](#_ENREF_7)). The estimated cost of lung cancer screening in the US is up to $2 billion/y. The subsequent management of IPNs, requiring serial CT scans, and sometimes invasive biopsies or surgical resection, is estimated to be many times greater, perhaps as much as $240,000 per additional lung cancer death avoided, when allowing for the costs of no screening and overdiagnosis ([8](#_ENREF_8)).

**Disease Management:** One management option for newly diagnosed indeterminate lung nodules is “watchful waiting” to see if the nodule increases in size over a two-year period, with the obvious caveat that during this time cells from a malignant nodule could metastasize, resulting in the lost chance of a cure. The other option, proceeding to tissue diagnosis at the time of discovery, is often inappropriate for several reasons. While bronchoscopy with brush cytology or transbronchial biopsy may be useful if the lesion is 2 cm or larger in size, the reported diagnostic yield of bronchoscopy for a solitary pulmonary nodule varies widely in the literature (20-80%) and depends on the size of the nodule, the incidence of malignancy in the study population, and the skill of the operator. Transthoracic fine needle aspiration biopsy can also be considered for lung nodules, but is most useful in nodules with a diameter of at least 2 cm. False negative results, occurring in up to 30% of the patients, remain a serious clinical problem. Unfortunately, the success rate of diagnosis is inversely related to nodule size, and yet the greatest hope for cure lies in detecting cancer as early as possible, presumably in smaller nodules. Thus, a definitive biopsy often requires surgical resection, *via* video-assisted thoracoscopic surgery (VATS) or open thoracotomy, since less invasive techniques have unacceptably high false negative rates ([9-11](#_ENREF_9)).

**Therapy:** Resection of early stage disease remains the only proven means to improve long-term survival from lung cancer. Although the smoking rate in the US is decreasing, it remains unacceptably high. The risk of lung cancer accordingly will remain elevated for many years due to smoking prevalence, general demographics, and potential exposure to other inhaled carcinogens ([1](#_ENREF_1)). Additionally, now that lung cancer screening of high risk individuals is adopted in the US, the capacity of the US Healthcare System is severely strained to meet demand. Nonetheless, low-dose annual CT screening for lung cancer in high-risk patients is the only proven means to reduce lung cancer deaths, with a 20% reduction in cancer-specific mortality ([4](#_ENREF_4)).

The US Preventative Services Task Force has endorsed LDCT lung cancer screening for high risk patients as an effective means of reducing lung cancer deaths, with a “B” level recommendation, equal to that of screening mammography. Thus, most US medical facilities already offer low-dose lung cancer screening programs ([12](#_ENREF_12)), and both medical and public advocacy societies have endorsed lung cancer screening, including the American Cancer Society, American College of Chest Physicians, American College of Radiology, American Society of Clinical Oncology, the National Comprehensive Cancer Network (NCCN), the American Lung Association and others ([13](#_ENREF_13)). In 2015, the Center for Medicare and Medicaid Services ([14](#_ENREF_14)) began to reimburse LDCT in high-risk patients within the Medicare population. It is estimated that approximately four million Medicare beneficiaries fit the eligibility criteria for screening, which includes adults age 55 to 74 who have a thirty pack/year smoking history and are either currently smoking or have quit in the past 15 years ([14](#_ENREF_14)). While the specific number of lives saved annually in the US by lung cancer screening will be affected by the adherence to the screening protocol, prevalence of lung cancer in the local screened population, and the effectiveness of associated smoking-cessation programs, the number is estimated to be about 12,000 annually ([15](#_ENREF_15)). In addition, under the Affordable Care Act, private medical insurance is required to cover lung cancer screening with no co-pay as of 2015. Accordingly, the number of new IPNs discovered annually in the US will increase several fold, reaching into the millions. Clearly the management of IPNs in the US population is a complex and resource-intensive task. As the US Healthcare System increasingly adopts lung cancer screening, improved means of managing IPNs will be urgently needed.

**Positron emission tomography (PET) imaging in lung cancer:** Imaging with computed tomography (CT) (either at a single time point or serially over time) or with positron emission computed tomography combined with CT (PET/CT) can help to discriminate between benign and malignant nodules, yet the best image-based methods are also inexact, with both false positive (FP) and false negative (FN) results leading to unnecessary surgery or missed early diagnosis, respectively. Collective experience with the most widely used PET tracer in oncology, 2-deoxy-2-(^18^F)-fluoro-D-glucose (^18^F-FDG), a probe that measures glucose utilization, has been variable. ^18^F-FDG PET possesses limited ability to discriminate between benign and malignant lung nodules less than 1 cm in diameter, as well as limited value in relatively quiescent lung cancers, such as well-differentiated adenocarcinoma or typical carcinoid tumors ([16](#_ENREF_16)). The sensitivity of PET scanning for small lesions drops significantly to around 60% ([17](#_ENREF_17)). In lesions smaller than 1 cm, only very intense ^18^F-FDG uptake is clearly perceived. Nomori, *et al.* ([18](#_ENREF_18)), examined 136 nodules smaller than 3 cm in diameter. All 20 nodules smaller than 1 cm were negative on standard-of-care ^18^F-FDG PET, eight (40%) of which were malignant. This problem is compounded by false positive (FP) uptake of ^18^F-FDG in IPNs. Much of the US population lives in areas with endemic fungal disease, such as histoplasmosis, coccidioidomycosis and cryptococcus. Vanderbilt University Medical Center (VUMC) is in the Ohio/Mississippi river valley complex, where coccidiomycosis and histoplasmosis are particularly common. Other large segments of the US population are in other areas of endemic fungal disease, such as the entire southwestern US, including southern California. In our prospective analysis of 211 patients referred to our thoracic oncology, thoracic surgery and/or pulmonary medicine services, the sensitivity of ^18^F-FDG PET/CT was good (92%) but the specificity was only 40%, with 60% of benign nodules having FP uptake ([19](#_ENREF_19)). Nearly identical results were reported from another location in the Ohio/Mississippi river valley complex by Croft, *et al.* at the University of Iowa ([20](#_ENREF_20)).

Highly proliferative cells, as found in most lung cancers, utilize Warburg metabolism to both provide energy and building blocks needed for cellular proliferation. This is an effect shared with all proliferating eukaryotic cells, but particularly expressed in aggressive malignancies, providing the target of metabolism exploited with ^18^F-FDG PET/CT. Unfortunately, other non-malignant cells with high glucose consumption, such as macrophages, particularly found in granulomatous nodules, also have high uptake of ^18^F-FDG ([21](#_ENREF_21)). These two factors often lead to the “false positive” uptake on ^18^F-FDG PET/CT scans in areas of endemic fungal and other granulomatous nodules ([22](#_ENREF_22)). This, of course, explains the high sensitivity of ^18^F-FDG PET/CT imaging of IPNs, along with its poor specificity (~40%), observed in many areas of the US.

Patients with newly discovered IPNs (often by “accident” when an x-ray or CT scan is performed for an unrelated reason) may be at high risk for lung cancer. Patients with IPNs or lung cancer typically have a significant smoking history, are often current smokers, and commonly have other tobacco-related co-morbidities such as COPD, emphysema and/or cardiovascular disease that greatly impair their functional status and confer high risk for interventional procedures needed to establish a definitive diagnosis.

Differentiation of a benign from a malignant lung nodule is imperfect at the time of IPN discovery. No combination of CT characteristics can definitively determine if a given IPN is benign or malignant ([23](#_ENREF_23), [24](#_ENREF_24)). While ^18^F-FDG PET/CT is helpful, it can be confounded by infection/inflammation, small nodule size, metabolically indolent cancers, and ground-glass or part-solid nodules. Moreover, in much of the United States, a variety of fungi and other granulomatous processes confound CT further, with most of the tracer uptake in active granulomatous nodules relegated to inflammatory stroma. Therefore, an improved means to discriminate benign from malignant IPNs at the time of discovery is badly needed.

Other PET probes have been tried, particularly in animal models of lung cancer, but none match the superior sensitivity of ^18^F-FDG. Given the life-threatening nature of lung cancer, and the number of IPNs seen on CT, we have a great need to improve upon the 40% specificity of ^18^F-FDG PET/CT for diagnosis of lung nodules in regions of endemic granulomatous infections, yet without sacrificing ^18^F-FDG’s ~90% sensitivity, particularly in the context of nationwide lung cancer screening.


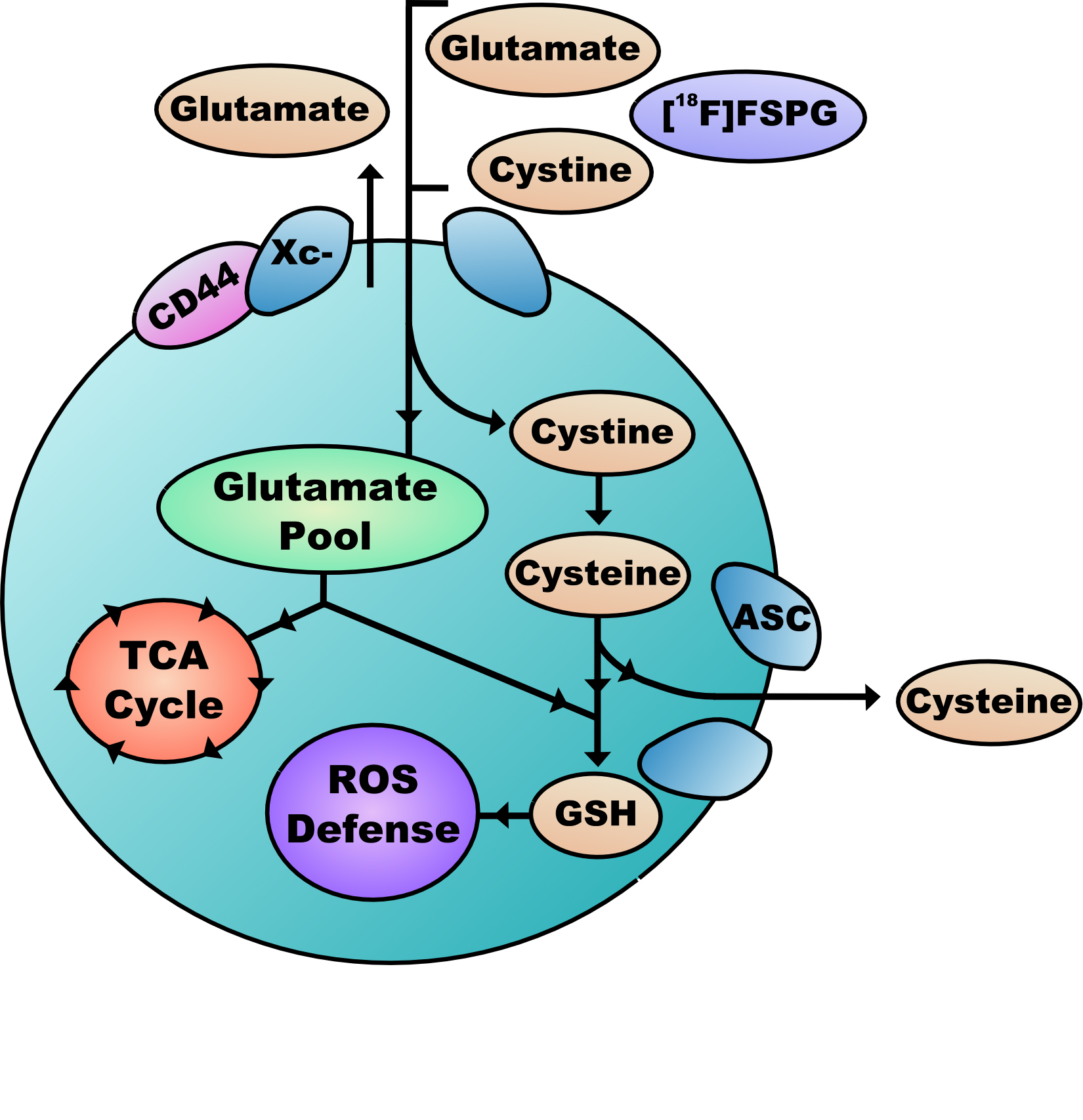


**Figure 1. Biological rationale for PET imaging of tumor cell metabolism.** Role of glutamate (*top*) in tumor cell metabolism.

**Figure 1. A Diagnostic PET Tracer of Cancer Cell Metabolism (^18^F-FSPG): Tumor cells adopt distinct metabolic pathways to ensure a sufficient supply of energy and building blocks for growth and proliferation. There is emerging evidence for the existence of a tumor-specific truncated tricarboxylic acid cycle (**[**25**](#_ENREF_25)**) associated with a high rate of lipogenesis and glutaminolysis from glutamine and glutamate (**[**26**](#_ENREF_26)**). As a result of the truncated TCA cycle, glutamine and glutamate are often major metabolic substrates of tumor cells in parallel or even instead of glucose-derived metabolites like pyruvate. Targeting those tumor-specific pathways with ^18^F-labeled tracers represents a promising approach for improved PET imaging of tumors.**

(*S*)-4-(3-[^18^F]-fluoropropyl)-L-glutamic acid (^18^F-FSPG) is a new ^18^F-labeled radiotracer designed for positron emission tomography (PET) imaging of tumors (**Fig. 1**). ^18^F-FSPG (originally [^18^F]BAY 94-9392) is an ^18^F-labeled glutamic acid derivative originally identified to target tumor-specific adaptations of intermediary metabolism. ^18^F-FSPG is specifically taken up *via* the system x_C_^-^ transporter, a glutamate-cystine exchanger (SLC7A11/SLC3A2 heterodimer) that transports amino acids L-glutamate and L-cystine (Cys-S-S-Cys) into the cell. The release of highly abundant intracellular glutamate is the driving force for efficient uptake of cystine and ^18^F-FSPG. Isotopic dilution of ^18^F-FSPG in a large intracellular glutamate pool leads to efficient trapping of the tracer, with no further metabolism of the tracer observed. Intracellularly, cystine is rapidly reduced to two molecules of cysteine (Cys-SH) and represents the rate-limiting precursor for glutathione ([27](#_ENREF_27)) biosynthesis. Redox maintenance and a constant supply of GSH are especially important in tumor cells as protection against reactive oxygen species (ROS) and for tumor survival ([28](#_ENREF_28)). As a result, many tumor cells possess a survival advantage over normal cells because they express a higher expression of the x_C_^-^ transporter, accumulate higher levels of L-cysteine and L-glutamate, and thus maintain high levels of GSH to detoxify ROS efficiently ([29](#_ENREF_29)).

In contrast to the modified intermediary metabolism often found in tumors, infection and inflammation related processes are primarily dominated by a high rate of glycolysis and/or glutaminolysis. Infiltrating macrophages and most other inflammatory cells do not proliferate at the target site, and thus depend mainly on glucose or glutamine to meet their bioenergetic requirements. ROS and redox-balancing processes are also important in these settings, wherein system x_C_^-^ may also play a role. However, the absence of an intracellular glutamate pool should not lead to high transporter activity and/or tracer retention as observed in tumor cells. This needs to be investigated in more detail and will be also part of this study to gain further insight for future applications of ^18^F-FSPG as a targeted cancer-imaging agent in the clinic.

Preclinical PET imaging studies with ^18^F-FSPG indicated superior tumor imaging when compared with ^18^F-FDG with rapid clearance from the blood and whole body leading to a low background from healthy organs and tissues. For example, a high uptake and retention of ^18^F-FSPG in NCI-H460 lung cancer cells was observed *in vitro* and *in vivo*. Other lung tumor cells lines (A549, NCI-H322, NCI-292) were studied with similar results ([26](#_ENREF_26)).

Furthermore, ^18^F-FSPG was tested in several inflammation models. In a combined tumor/inflammation model, where inflammation was induced by turpentine oil, ^18^F-FSPG showed superior differentiation of tumor tissue from inflammatory lesions compared to ^18^F-FDG ([26](#_ENREF_26)). Other inflammation models were studied but the results are not yet published (personal communication Piramal Imaging). This included also inflammation models using Freund’s complete adjuvant, a colitis model, and a wound healing/scar tissue model that showed similar results to the turpentine oil model and were all negative for ^18^F-FSPG uptake. Only a rodent model of lymphadenitis and one mouse with incidental histiocytosis were observed to be positive for FSPG. More research is needed to understand what led to increased ^18^F-FSPG uptake and retention in this situation, though sarcoidosis and active tuberculosis do demonstrate strong ^18^F-FSPG uptake([30](#_ENREF_30)).

Toxicology and safety pharmacology studies with the ^19^F-labeled drug substance (“cold” fraction of the active compound) and decayed drug formulation (containing by-products and impurities) confirmed that ^18^F-FSPG can be used in clinical trials. Furthermore, the calculated effective radiation dose (ED) dose is within acceptable limits for PET imaging in humans (*see below*).

In a pilot clinical study of lung and breast cancer by Baek *et al.* ([31](#_ENREF_31)) a high tumor detection rate was observed for NSCLC but not for breast cancer. A positive ^18^F-FDG-PET was used as the inclusion criterion. All 10 ^18^F-FDG-positive lung cancer patients showed ^18^F-FSPG uptake. Interestingly, Baek *et al.* reported that elevated glycolytic activity and uptake of ^18^F-FSPG appeared to be correlated in lung cancer, while a similar relationship was not observed in breast cancer ([31](#_ENREF_31)). Immunohistochemical (IHC) analyses on these subjects’ pathology samples showed significant correlation between ^18^F-FSPG uptake and protein expression of both the SLC7A11 subunit of system x_C_^-^ and the stem cell marker CD44. In breast tumor samples specifically, IHC showed that absence of CD44 correlated with low signal from ^18^F-FSPG-PET, even if the SLC7A11 subunit was present, indicating possible importance of CD44 co-expression for system x_C_^-^ function. The standardized uptake values of ^18^F-FSPG for non-small cell lung cancer (NSCLC) averaged 7.6 (+/- 7.3), *versus* 13.0 (+/- 6.7) for ^18^F-FDG. While the average uptake values for ^18^F-FSPG were lower than for ^18^F-FDG, Baek, *et al*, ([31](#_ENREF_31)) demonstrated that the background signal was lower for ^18^F-FSPG, leading to comparable tumor to background ratios with ^18^F-FDG.

In a pilot clinical study ^18^F-FSPG PET was investigated in patients with inflammatory lesions and compared with ^18^F-FDG. The SUV_max_ of all lesions was significantly lower (~3.6 times) for ^18^F-FSPG compared to ^18^F-FDG (SUV_maxFSPG_: 1.8 *versus* SUV_maxFDG_: 6.5), with the exception of sarcoid lesions; rapid washout was also observed. Such incidences in our target population is low and it is expected that these are unlikely confounders on the differential diagnosis.

The SUVs for ^18^F-FSPG in NSCLC were higher compared with other radiopharmaceuticals in this setting: ^18^F-Fluorothymidine (^18^F-FLT) showed SUV values of 3.54 (+/- 1.98) ([32](#_ENREF_32)). Hustinx *et al.* ([33](#_ENREF_33)) reported on the use of another amino acid (2-^18^F-fluoro-L-tyrosine, ^18^F-TYR) for imaging in cancer, including lung cancer, but with only 6 out of 11 lung cancer patients demonstrating similar staging compared to ^18^F-FDG, with the ^18^F-TYR SUV value for lung cancer being approximately 2.0. Beer *et al.* ([34](#_ENREF_34)) investigated a PET imaging probe for angiogenesis, ^18^F-galacto-RGD, and compared it to ^18^F-FDG in PET/CT imaging of 18 patients with primary or metastatic cancer, 10 of whom had NSCLC, with analysis of uptake in all primary tumor and metastatic lesions. There was no correlation between the uptake values of ^18^F-galacto-RGD and ^18^F-FDG, with the latter probe being clearly superior. Thus, while radiolabeled amino acids have proven limited usefulness for PET imaging of neoplasms in the lung, prior attempts to use amino acids and other PET imaging agents beyond ^18^F-FDG are, until now, of limited clinical value.

In summary, ^18^F-FSPG is a novel PET tracer to assess the activity of system x_C_^-^ and has shown promise as a cancer-imaging probe in a variety of preclinical and clinical settings. A favorable biodistribution pattern and a high lesion detection rate previously seen in rodents ([26](#_ENREF_26)) was confirmed in patients with different tumor types. Phase I clinical trials have been completed assessing the safety and tolerability as well as the tumor targeting potential and tumor accumulation of ^18^F-FSPG in healthy volunteers and patients with various cancers. To date, ^18^F-FSPG has been evaluated in patients with breast and lung cancer, where the tracer exhibited a promising detection rate of tumors with x_C_^-^ transporter activity ([31](#_ENREF_31)). Ten of 10 ^18^F-FDG-positive lung cancer patients showed ^18^F-FSPG uptake. Interestingly, Baek *et al.* reported that elevated glycolytic activity and uptake of ^18^F-FSPG appeared to be correlated in lung cancer, but a similar relationship was not observed in breast cancer ([31](#_ENREF_31)).

**Benefit of this research to patients**

This research may impact patient care in a number of positive ways. Results from this study have the potential to establish a new role for non-invasive molecular imaging in distinguishing benign indeterminate pulmonary nodules from lung cancer. The benefits potentially include reducing the need for some follow-up CTs, unnecessary invasive biopsies with attendant morbidity and rare mortality, reducing patient anxiety, and an overall decrease in wasted healthcare spending. If ^18^F-FSPG PET is shown to be more specific for lung cancer compared to ^18^F-FDG PET for diagnosis of IPNs, ^18^F-FSPG PET may enable improved detection of smaller lesions and improved discrimination of benign from malignant lesions compared to the current standard of care assessments.

**2. Objectives, Rationale, and Specific Aims**

This is a hypotheses-driven prospective clinical trial of an emerging, molecularly targeted PET reagent, ^18^F-FSPG.

*The objective is to test the hypothesis that ^18^F-FSPG PET/CT will provide improved accuracy in diagnosis of IPNs compared to the current PET/CT imaging standard-of-care with ^18^F-FDG.*

*Our rationale is that ^18^F-FSPG PET/CT may yield improvements in discrimination of tumor from benign IPNs.*

There are three Aims:

**Specific Aim 1: To compare the imaging characteristics of ^18^F-FSPG PET/CT with standard-of-care, ^18^F-FDG PET/CT.**

*Hypotheses: ^18^F-FSPG will accumulate in lung tumors with equal or improved sensitivity and increased specificity over SOC ^18^F-FDG PET/CT.*

*Approach: We will determine and compare the accuracy in discrimination between benign and malignant lung nodules detected by CT between these two ^18^F imaging probes for the diagnosis of lung cancer in a population whose IPN’s are 7-30 mm in maximum diameter, or in lung masses not yet biopsied that are more than 3 cm in diameter.*

**Specific Aim 2: To compare the imaging characteristics of ^18^F-FSPG PET/CT to standard-of-care ^18^F-FDG PET/CT in patients with newly diagnosed lung cancer.**

*Hypotheses: ^18^F-FSPG will provide improved baseline diagnosis and staging of lung cancer relative to SOC PET/CT using ^18^F-FDG.*

*Approach: We will determine whether ^18^F-FSPG PET/CT is superior to ^18^F-FDG PET/CT for baseline diagnosis and staging of lung cancer, and whether the biodistribution of ^18^F-FSPG varies in various histological subtypes, grades, and stages of lung cancer.*

**Specific Aim 3: To determine whether ^18^F-FSPG uptake in lung cancer can be predicted based on correlation with CD44 and x_C_^-^ expression in surgical pathology specimens.**

*Hypotheses: ^18^F-FSPG PET will correlate with x_C_^-^ and CD44 immunoreactivity in tumor and non-tumor (IPN) tissues.*

*Approach: Core or surgical biopsies and/or autopsy specimens will be obtained from, following standard-of-care for subjects, in Specific Aims 1 or 2. This profiling will allow us to determine whether FSPG uptake may potentially distinguish a subclass of lung cancers with different clinical behavior, e.g. possibly identifying very high risk disease even in early stage lung cancer****.***

**3. Animal Studies and Previous Human Experience**

Published reports note that ^18^F-FSPG has been evaluated previously in rodents and humans ([31](#_ENREF_31), [35-37](#_ENREF_35)) . To date, ^18^F-FSPG has been evaluated with promising results in over 70 patients with lymphoma and lung, breast, head and neck, brain, and prostate cancers, where the tracer exhibited a promising detection rate of tumors with x_C_^-^ transporter activity. In a pilot study conducted at Seoul, South Korea, Baek *et al.* reported that elevated glycolytic activity and uptake of ^18^F-FSPG appeared to be correlated in lung cancer, but a similar relationship was not observed in breast cancer ([31](#_ENREF_31)). In preclinical studies, ^18^F-FSPG exhibited greater tumor avidity compared to inflammation in a combined tumor-inflammation model in rats ([26](#_ENREF_26)). In another publication from Korea, ^18^F-FSPG was evaluated to be useful in patients with HCC who exhibited a range of ^18^F-FDG PET results ([35](#_ENREF_35)). To date, in human studies, ^18^F-FSPG PET has been well tolerated, with no reported safety concerns ([35](#_ENREF_35)).

**4. Patient Eligibility & Inclusion / Exclusion Criteria**

Participants will be recruited by Vanderbilt University Medical Center and Tennessee Valley Health System Nashville (TVHS) study team members. Patient history will be reviewed for inclusion/exclusion criteria and informed consent obtained as described below.

Enrollment will be open to all patients of both genders with the above diagnoses described in the Specific Aims, with the following inclusion/exclusion criteria.

**A. Inclusion Criteria**

1. Adult patient (age 40 - 80).
2. An indeterminate pulmonary nodule (IPN) (7–30 mm diameter) on CT, or an indeterminate lung mass (> 30 mm diameter), without prior examinations that establish that the lesion has been stable for two or more years.
3. Untreated.

*OR*

4. An adult patient with a newly diagnosed, untreated primary lung cancer diameter 7 mm or more.

*AND*

5. The patient must provide informed written consent, which will include a layman’s explanation of the estimated amount of additional radiation that the patient will receive from the investigational PET/CT scan using ^18^F-FSPG.

6. The patient must agree at the time of study entry to undergo clinically indicated biopsy(ies) or a 24-month period of follow-up, as needed, to resolve the etiology of their IPN(s) or lung mass(es).

**B. Exclusion Criteria**

1. Pregnant or lactating patients will be excluded, as will females of childbearing potential who refuse to undergo a serum or urinary beta-HCG pregnancy test the day of either the ^18^F-FSPG or the ^18^F-FDG PET/CT scans, in accordance with the standard policy of the Medical Imaging Service at our facility. Women who have experienced 24 consecutive months of amenorrhea, have reached at least 60 years of age, or have had a tubal ligation or hysterectomy documented in their medical records are considered not to be of childbearing potential for the purposes of this protocol.
2. Patients with a body weight of 400 pounds or more or a body habitus or disability that will not permit the imaging protocol to be performed, due to the compromise in image quality on both CT and PET/CT. If the standard-of-care ^18^F-FDG/PET was of diagnostic quality as determined by the official clinical interpretation, then this will be presumptive evidence that the patient’s body habitus and/or disabilities should not prevent a diagnostic quality ^18^F-FSPG PET/CT scan, either.
3. A recognized active lung infection (this will confound the standard-of-care ^18^F-FDG PET/CT scan).
4. Previous systemic or radiation treatment for cancer of any type within 1 year.
5. For patients who do not have a tissue diagnosis:
   1. Non-oncologic severe co-morbidities suggesting a life span of less than two years if not treated, as determined by the potential subject’s treating physician.
   2. If severe co-morbidities are present, the treating physician should indicate that a life span of 2 years is expected if treatments are effective.
   3. This exclusion is to prevent loss of the needed 2 year CT follow-up to establish a benign diagnosis for lesions lacking tissue diagnosis if extremely fragile subjects are enrolled and then experience an untimely, unrelated death.

**5. Enrollment/Randomization**

We propose a three (3) year funded prospective cohort with consecutive subject enrollment throughout each year. We will enroll subjects referred to the Oncology, Thoracic Surgery or Pulmonary Medicine Services at Vanderbilt University Medical Center and Tennessee Valley Healthcare System Nashville. We will allow a 2-year follow-up of all subjects to document stability by CT for IPNs that do not have a tissue diagnosis (stability on CT for two years of an IPN or mass is accepted presumptive evidence of benignity). Follow-up will be from the day of the baseline CT, and may be extended later if needed. At VUMC, we see about 750 patients with newly diagnosed IPNs annually, and about 400 patients with newly diagnosed lung cancer. Accordingly, given the high prevalence of granulomatous IPNs in our geographic region, we are well suited to perform the work we have described.

We will perform an analysis to correlate the ^18^F-FSPG PET/CT findings with ^18^F-FDG PET/CT and the surgical pathology results for the patients who have tissue correlation. Each subject will receive the “standard-of-care” ^18^F-FDG PET/CT as indicated for patients at high risk for lung cancer who have an IPN or lung mass meeting our size criteria, and will also receive the investigational ^18^F-FSPG PET/CT, with no intervening treatment of the target lesion(s). After enrolling 30 subjects, we will close enrollment but continue the study for data collection and analysis until the funding period ends and/or sufficient follow-up for diagnosis (up to 2 years) has occurred, and report our results in the peer-reviewed literature.

Should we achieve clear statistical superiority of ^18^F-FSPG PET/CT over ^18^F-FDG PET/CT before that time, we will pursue a larger trial to offer improved benefit of our patients for the diagnosis of IPNs and/or staging of lung cancer (*see* Go/No-Go criteria in Study Design). While 2 years’ stability in nodule size is the “gold standard” for CT, well over 90% of cancers demonstrate growth on CT within one year. We will also perform the research outlined in Specific Aim 3 on tissue specimens obtained from our subjects in Specific Aims 1 and 2. If we find significant added value from the use of ^18^F-FSPG in either IPNs or lung cancer, we will propose further investigations.

Final diagnosis will be based on tissue biopsy (surgical or *via* a high-quality core biopsy) or negative (no growth) two-year follow-up with CT. We will assign patients with IPNs into the “benign” category if their IPNs remain stable for two or more years, decrease in size, or resolve during CT follow-up, performed in accordance with Fleischner Society guidelines.

**Patient Registration**

All patients MUST be registered with the Vanderbilt-Ingram Cancer Center (VICC) prior to the start of research procedures for all sites.

Patients will be centrally registered with the Vanderbilt-Ingram Cancer Center (VICC) at study entry by emailing the Multi-Institutional Team at coordinating.center@vumc.org. At the time of registration, the following documents must be submitted:

- Copy of the patient’s signed and dated Informed Consent
- Patient Enrollment From
- Eligibility Checklist including source documents to verify eligibility

The VICC Coordinating Center will assign sequence numbers to all patients in screening. Sequence numbers will not be re-used if a patient screen fails.

**6. Data Management and Monitoring**

Participating institutions will be collaborating with Vanderbilt in patient accrual. Data will be collected using a centralized electronic case report form called ON-line Clinical Oncology Research Environment = Oncore (http://www.vicc.org/ct/research/oncore.php). Oncore is a highly secure, web based, cancer specific, and customizable system that provides fully integrative clinical data management and study administration capabilities developed in an ongoing collaborative effort with NCI designated Comprehensive Cancer Centers. It fully integrates study administration functionality including protocol tracking, patient registration, NCI reporting, review committee tracking, and SAE tracking, with clinical data management functionality including electronic case report forms (eCRF) design, clinical data capture, protocol and regulatory compliance monitoring. Also the system is capable in storing basic protocol information (e.g., IRB approval dates, dates for annual renewals,) and clinical trials research data. Oncore allows the investigator to define specific protocol requirements and generate data collection forms. Creation of the data collection form is done with a single button click after the parameters of an individual protocol have been specified. Oncore permits specification of study protocols, management of patient enrollment, clinical data entry and viewing, and the generation of patient or study-specific reports based on time stamping. OnCore is embedded with a comprehensive domain repository of standard reference codes and forms to promote standardization. The sources for the repository include CDUS, CTC, CDEs from NCI, ICD, MedDRA and various best practices from contributing NCI-designated Comprehensive Cancer Centers. OnCore provides several reporting features specifically addressing NCI Summary 3 and Summary 4 and other reporting requirements. Data may also be exported in a format suitable for import into other database, spreadsheets or analysis systems (such as SPSS). This system will be used to manage all VICCC clinical trials data. OnCore is maintained and supported in the VICC Clinical and Research Informatics Resource.

Specified members at each participating site will submit all pertinent regulatory documents to the Coordinating Center, who will store it in a secure location.

As the Coordinating Center, Vanderbilt has responsibilities to health authorities to take all reasonable steps to ensure the proper conduct of the study as regards to ethics, protocol adherence, integrity, validity of the data recorded on the CRFs, and adherence to regulations regarding Good Clinical Practice (GCP) and the protection of human subjects.

In accordance with applicable regulations, GCP, and Coordinating Center procedures, sites will be contacted prior to the start of the study to review with the site staff the protocol, study requirements, and their responsibilities to satisfy regulatory, ethical, and Coordinating Center requirements.

During the course of the study, the Coordinating Center will routinely monitor sites for protocol compliance, compare CRFs with individual subjects’ original source documents, assess drug accountability, and ensure that the study is being conducted according to the pertinent regulatory requirements. The review of subjects’ medical records will be performed in a manner to ensure that subjects’ confidentiality is maintained. Monitoring visits will primarily be conducted remotely, and sites are required to provide the appropriate source documentation in order to allow for proper oversight per GCP. Investigators must agree to cooperate with the Coordinating Center to ensure that any problems detected are resolved.

The VICC DSMC meets on a quarterly basis and ad hoc to discuss data and safety monitoring of clinical trials and to oversee the VICC DSMP. Internal audits for compliance with adverse event reporting, regulatory and study requirements, and data accuracy and completion are conducted according to the VICC DSMP according to study phase and risk. The committee reviews all serious adverse events (SAE) on Vanderbilt sponsored investigator-initiated studies on a quarterly basis and provides DSMC SAE review reports to the Vanderbilt IRB.

**7. Study Procedures & Data Collection**

**Baseline Imaging: CT**

Each patient will have a diagnostic CT within 180 days prior to enrollment (as part of the standard of care) to define the pathologic conditions necessary to be enrolled, and be untreated for the IPN or lung cancer for which the patient is being evaluated. These prior scans must be immediately available to the investigators for review. Outside CT scans found to be of good quality will be acceptable if they are available in native (uncompressed) DICOM format.

**Imaging Protocol: ^18^F-FSPG (Figure 1)**

Each patient will undergo an SOC whole-body (vertex to mid thighs) ^18^F-FDG PET/CT scan within 60 working days of an ^18^F-FSPG PET/CT scan for these first 60 enrollees, with no intervening cancer treatment of the target lesion (limited biopsies or resections are allowed). A minimum of 12 hours must lapse between SOC (^18^F-FDG) and ^18^F-FSPG PET/CT scans. Standard of care procedures performed prior to consent but within the protocol defined windows for each assessment can be used for study purposes. All research only procedures must be performed after the consent date.

Eight (8) subjects with either indeterminate nodule(s) or thought or known to have newly diagnosed lung cancer, will receive an ^18^F-FSPG PET scan (emission only), acquired dynamically after injection over the nodule/mass of interest for 60 minutes, in order to characterize the time activity curves of ^18^F-FSPG. These 8 subjects will then have a short break (bathroom, stand up) and then receive a PET/CT scan from the vertex to the mid-thighs. This final body PET/CT covers the same anatomic range as the SOC ^18^F-FDG PET/CT, and will be compared closely to the ^18^F-FDG PET/CT to determine if there is added value of the ^18^F-FSPG PET/CT. Once the dynamic PET/CT scans with ^18^F-FSPG in the initial 8 subjects are obtained, the remainder of subjects will have a scanning protocol from vertex to mid-thighs about 60 minutes after ^18^F-FSPG injection, similar to the protocol for standard of care ^18^F-FDG PET/CT.

The third year will be used, if needed, to obtain at least two years of CT follow-up for patients with IPNs who chose serial follow-up CT scans over a tissue diagnosis (two years is required by Fleischner Society Guidelines to prove stability/benignity for solid nodules), though over 90% of malignant IPNs will demonstrate growth within one year by CT.

Figure 1: Flow Schema of Imaging:


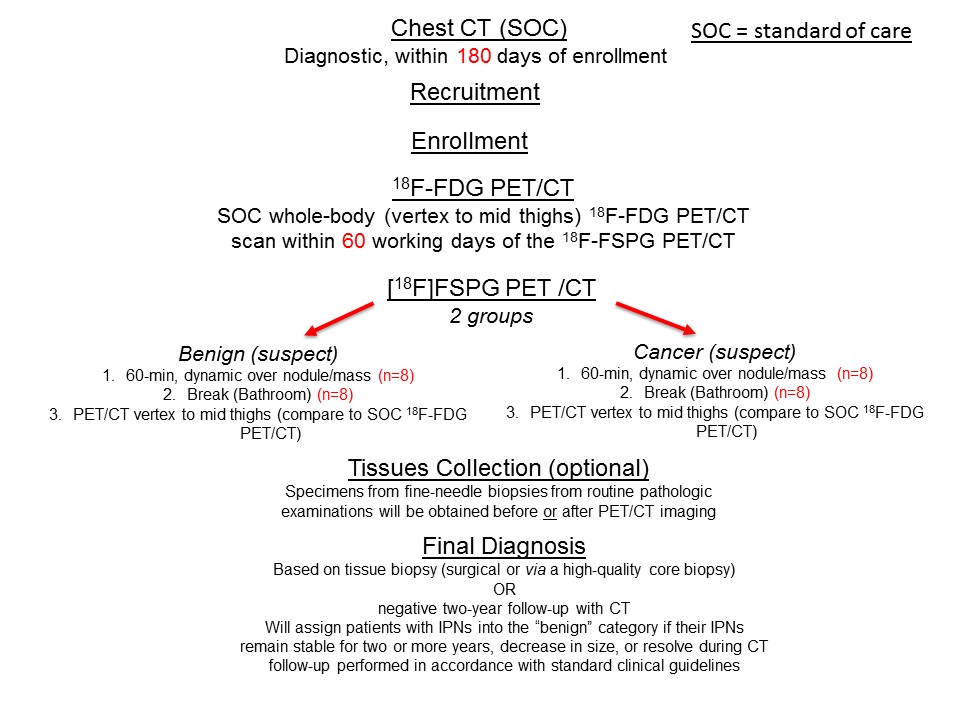


*Optional tissues collection noted in Figure 1 are part of separately enrolled study and not called for on this protocol.

It is possible that we will complete enrollment of sufficient patient numbers prior to the end of year 1, in which case we will continue the clinical follow-up period for the remainder of the proposed investigational period until the end of the proposed funding or until (if sooner) we achieve clear statistical advantage of ^18^F-FSPG over ^18^F-FDG PET/CT for the diagnosis of IPNs and/or staging of lung cancer. If statistical superiority of ^18^F-FSPG PETCT is established, we will initiate a larger trial (additional 40 subjects) for the use of ^18^F-FSPG PET/CT in the diagnosis if IPNs (Specific Aim 1) and/or evaluation of lung cancer (Specific Aims 2 and 3) (*see* Go/No-Go criteria in Study Design).

At Vanderbilt University Medical Center, the human PET scanners are integrated PET-CT systems. Therefore, a whole body transmission CT scan without contrast is performed on all patients who undergo a PET scan. The transmission CT scan is used to correct the PET images for attenuation and provide CT images for anatomical mapping. The ^18^F-FSPG will be prepared by the Vanderbilt University Medical Center Radiochemistry Core Laboratory under IND 124202 (H. Charles Manning, PI). For consistency, patients will fast at least four (4) hours and the blood glucose concentration (“finger-stick” in the PET area) will be determined prior to injection of all radiopharmaceuticals.

Analysis of ^18^F-FDG and ^18^F-FSPG PET/CT images will be performed independently by at least 2 physicians at VUMC who are experienced in PET/CT interpretation with a third physician used for consensus if there is a disagreement as to interpretation. The images will be interpreted with US FDA software on VUMC’s GE Healthcare AW 2 and/or Xeleris 3 workstations.

###

**Radiotracer Production**

^18^F-FSPG is obtained by radiolabeling of the protected precursor di-*tert*-butyl (2*S*,4*S*)-2-(3-((naphthalen-2-ylsulfonyl)oxy)propyl)-4-(tritylamino)pentane-dioate) (PI-021) with [^18^F]fluoride. After acidic deprotection, the tracer is purified over cartridges and finally formulated for i.v. injection by passing the solution through a 0.22-μm sterile filter. It has a half-life of 109.7 minutes. The synthesis is performed within an automated synthesis module in a lead-shielded hot cell. The production methods, sterile filtration, and formulation are all carried out under Vanderbilt-held IND 124202, and allow for the production of a sterile and pyrogen-free solution ready for injection.

**^18^F-FSPG PET Procedure and Dynamic Data Analysis**

For ^18^F-FSPG and standard of care ^18^F-FDG PET/CT, imaging is described above. The dynamic analysis of the first 60 minutes of emission-only imaging will be analyzed as described below, in the initial 8 subjects, and used to determine the optimal imaging protocol, via analysis of time activity curves, for the investigational radiopharmaceutical to evaluate indeterminate lung nodules and lung cancer. This analysis will be performed at the Vanderbilt University Institute for Imaging Sciences (VUIIS), Manning laboratory, on dedicated workstations with appropriate software tools in place.

**Clinical Data Analysis**

The research PET/CT studies will be interpreted independently by two experienced nuclear medicine (NM) physicians blinded to each other’s interpretation. These NM physicians will render “un-blinded” interpretations with access to conventional imaging studies and all other relevant information available in the patients’ medical records. The final PET/CT interpretations (^18^F-FSPG; ^18^F-FDG PET/CT, clinical CTs and other relevant clinical imaging available for comparison) will then be compared to determine the impact of ^18^F-FSPG PET/CT on the final diagnosis, and specifically to determine if the ^18^F-FSPG PET/CT would have changed the diagnosis and/or stage of disease compared to the clinical SOC CT and/or ^18^F-FDG PET/CT. These results will be reported to the biostatistician who will compile the data for estimation for analysis (*see* Statistical Consideration Section).

At this time, the investigational data will not be used to direct care (e.g. influence treatment decisions) until we have sufficient knowledge of the usefulness of the data in this regard compared to standard of care procedures.

**Rationale for Size Criteria**

The rationale of a minimum diameter of 7 mm for IPNs and lung cancer for our investigation is based on the usual lower limit of detectability on PET imaging. While this is usually reported as 8-10 mm in the literature, much of this is based on older technology PET scanners. The size of 7 mm is also the minimum size in which evaluation of an IPN becomes technically feasible ([11](#_ENREF_11), [38](#_ENREF_38)) in terms of:

1) Number of patients to manage

2) Limited clinical means by which to obtain tissue diagnosis of such a small IPN

3) Risks/benefits of definitive evaluation of a nodule this small

While overall very safe, definitive evaluation of such small IPNs often requires a core biopsy *via* a transthoracic or transbronchoscopic biopsy, VATS wedge resection or even an open thoracotomy since fine-needle biopsies are often non-definitive. Given the co-morbidities frequently present in patients at high risk for lung cancer, the perioperative mortality is likely to be 1.0%. Since the majority of IPNs, even in high-risk patients, are benign, we must choose a minimum size threshold at which the potential benefits for the patient justify the pain, cost and risk of a definitive answer.

Henschke *et al.* ([39](#_ENREF_39)) recently addressed this in a retrospective review of 21,136 subjects enrolled in a low-dose CT lung cancer screening program performed between 2006–2010, in which malignancy rates of various size ranges of IPNs were evaluated. With a cut-off of 7 mm in diameter for IPNs, instead of 5 mm, was used, the percentage of patients with a “positive” screening CT at baseline for an IPN decreased from 16% (5 mm threshold) to 7.1% (7 mm threshold). When these patients were then evaluated for subsequent diagnosis, a delay in diagnosis of lung cancer by 9 months occurred in about 5% of patients using the threshold of 7 mm diameter as the minimum size for close interval follow-up, while the percent of “positive” screening exams decreased by over half (**Table 1**). Accordingly, though still debated, the definition of a “positive” CT screening examination for lung cancer will likely need to address practical, realistic management issues that will include the size of the IPNs to be definitively evaluated *versus* followed, particularly given the low but real morbidity/mortality associated with invasive procedures when the vast majority of even high-risk patients will have a benign IPN. Given these practical management results and the limits of resolution of current PET scanners, we have chosen the lower limit of size for our investigation of IPNs or lung cancer to be 7 mm.

**Table 1***

| IPN diameter (mm) defining a “positive” CT | 5 | 6 | 7 | 8 | 9 |
| --- | --- | --- | --- | --- | --- |
| Total percent of patients “positive” on CT | 16% | 10.2% | 7.1% | 5.1% | 4.0% |
| Patients (%) meeting diameter threshold with delay in cancer diagnosis of 9 months | 0% | 0% | 5.0% | 5.9% | 6.7% |

*Henschke *et al.* ([39](#_ENREF_39))

**Pathology Methods**

*x_C_^−^ and CD44:* To examine the correlation of accumulation of ^18^F-FSPG PET with x_C_^−^ and CD44 in lung tumors, tissues specimens from fine-needle biopsies obtained from routine pathologic examinations will be obtained before or after PET/CT imaging for immunohistochemistry (IHC) and Western blotting (WB) studies. For patients having surgical excision/biopsy after ^18^F-FSPG PET/CT, this tumor specimen will be used for further pathologic examination. Tissue processing and IHC analysis of formalin-fixed, paraffin-embedded tissue sections will be performed as detailed in our previous publications ([40](#_ENREF_40), [41](#_ENREF_41)). Briefly, 4-μm thick whole tissue sections were transferred onto poly-L-lysine-coated adhesive slides and dried at 74 °C for 30 minutes. After standard heat epitope retrieval for 1 hour in EDTA, pH 8.0, in the autostainer, the samples were incubated with antibodies for targeted epitopes. The level of expression of our proteins in both membrane and cytoplasm and in the membrane of malignant tumor cells will be examined by an experienced pathologist who is completely blinded to any patient and imaging information. The numerical results of the IHC will be semiqualitatively evaluated using a scale of 0, 1+ (weak), 2+ (medium), and 3+ (strong) with a sample being reported as positive if greater than 10% of the cells in the sample were positively stained. The correlation between the intensity of IHC staining and SUV_max_ of the corresponding lesion on the PET/CT will be assessed. This processing approach will allow for comparison of definite pathology and tracer imaging findings.

*TMAs:* Tumor and benign specimens will be assembled in Tissue Microarrays (TMAs) with associated clinical information. All TMAs will be paraffin-embedded blocks formed from cores derived from multiple paraffin-embedded tissue blocks, containing hundreds of cases in a single accessible block, fully annotated with clinical data elements covering patient demographics, past medical history, smoking history, details about the tumor diagnosis, stage, histology, imaging, and follow-up. TMA construction begins with a detailed plan and approval by the VUMC IRB and the VICC Lung SPORE PTIC Tissue Utilization Committee with successful data sharing agreements and off-site tissue banking waivers successful in the past. Slides corresponding to the tissue blocks of interest are reviewed by pathologist (Dr. Eisenberg) director for tumor identification and marking, and the block construction (Beecher Instruments, USA) is completed. To date, we have 26 lung-specific TMAs containing samples from approximately 750 patients with associated clinical data and including a variety of tumor types.

*Immunohistochemistry (IHC):* TMA tissue sections are evaluated for intensity and distribution of IHC staining. Scoring is performed by one or more pathologists, including the core Tissue Biorepository director, Dr. Rosana Eisenberg. The scoring system routinely used for evaluation of IHC staining is an index based on intensity and percent tumor positivity modeled after methods we have used extensively in the past ([40](#_ENREF_40)). In addition to the intensity and estimate of percent positive tumor cells, the cellular localization of staining is documented (nuclear, cytoplasmic, or membrane).

**8. Risks**

**Radiopharmaceutical**

The radiopharmaceutical (^18^F-FSPG) used in this study for PET scanning has been evaluated in humans previously ([31](#_ENREF_31), [35](#_ENREF_35)). No adverse side effects such as anaphylactic reactions, allergic reactions, or mortality or morbidity have been reported to the best of our knowledge. For a complete safety listing of ^18^F-FSPG, please refer to the Investigational Brochure (IB) of Piramal-held IND 121728. The total mass of radiopharmaceuticals administered in this study is in the sub-pharmacological amount (<100 micrograms), and thus is expected to have no physiologic effect on the patient or tumor. ^18^F-FSPG is produced by the Vanderbilt Radiochemistry Core Laboratory according the CMC section of Vanderbilt-held IND 124202 (Henry Charles Manning, PhD, PI) and meets all USP <823> requirements for a sterile, injectable PET radiopharmaceutical.

**Injection**

Risks and side effects related to the IV catheter may include discomforting pain at the site of injection, bleeding, and bruising.

**Reproductive Risk**

Patients should not be pregnant while on this study because the imaging agent being tested contains a small amount of radioactivity. This could affect an unborn baby. Female subjects of childbearing potential must have a negative serum or urinary pregnancy test within 24 hours of the proposed investigational PET/CT scan prior to injection of the investigational radiopharmaceutical in accordance with the policies of the Vanderbilt University Medical Center IRB and Department of Radiology.

**Dose Rationale**

A dose of 300 MBq (8.1 mCi) for ^18^F-FSPG studies was chosen in accordance with a typical radioactive dose of ^18^F-FDG used for oncological diagnostics (350 MBq for an adult). This dose is expected to provide suitable imaging contrast of the target structures.

**Radiation Safety**

Effective radiation doses for ^18^F-FSPG were calculated from biodistribution studies in mice and were determined in five healthy volunteers. Extrapolated effective doses from mice are 5.10 mSv for a male and 6.54 mSv for a female subject, assuming a patient dose of 300 MBq and a bladder-voiding interval of 45 min. In humans, an effective dose of 4.5 mSv was determined in healthy volunteers from an injection of 300 MBq/8.1 mCi of ^18^F-FSPG.

**Toxicity Studies**

The tracer (^18^F-FSPG) used in the study has been evaluated in humans previously without incident ([31](#_ENREF_31), [35](#_ENREF_35)). The evaluation of the toxicity of FSPG included an extended single-dose toxicity study in rats and in dogs and the evaluation of the genotoxic potential in a gene mutation assay (Ames test) and an assay on clastogenic effects (micronucleus assay *in vitro*). For a complete description of ^18^F-FSPG toxicity, please refer to the Investigational Brochure (IB) of Piramal-held IND 121728. Additionally, the effects of the impurities of the anticipated clinical kit formulation were evaluated in an extended single dose toxicity study over three days in rats with a decayed kit-formulation. All toxicological studies were performed according to GLP principles. Since these studies were intended to evaluate the chemical toxicity of FSPG and not the potential effects of the radiation, the non-radioactive ^19^F-labeled analogue was used for toxicological testing.

**9. Reporting of Adverse Events or Unanticipated Risks to Participants or Others**

Dr(s)., Shah, Manning, and attending nuclear medicine physician(s) will monitor the study progress and safety of the participating patients and will monitor for serious symptoms or toxicities on an ongoing basis. Vanderbilt’s Clinical Trials Shared Resources department will undertake reporting any significant adverse events to the IRB as required, to Piramal (until Piramal funding ceases), and to the IND holder, Dr. Manning. Dr. Manning and the study team will meet regularly, based on enrollment, to review and discuss any adverse events related to this study and report these to the FDA as required. Patients will be asked to report to the nurse any adverse symptoms that occur within 30 days of the research PET studies. Serious adverse events related to the study drug (^18^F-FSPG) or the performance of the PET/CT scan will be reported within three working days to the IRB and FDA as required, and non-serious adverse events at the time of continuing review. These adverse events will be recorded in the database maintained by the research nurse.

Adverse events for the purposes of this imaging trial are defined as any unexpected medical occurrence in a subject who receives ^18^F-FSPG. The event does not necessarily have to be causally related to the PET radiopharmaceutical to qualify as an adverse event. An adverse event can be any unfavorable or unintended signs, symptom or disease temporally associated with the injection of one of the radiopharmaceuticals, whether or not it is considered related to ^18^F-FSPG PET. Subjects will be monitored for adverse events during the actual imaging period and for up six half-lives following the research PET study. Since the investigational drug, ^18^F-FSPG, decays to background in 6 half-lives, these adverse events should occur within 660 minutes (11 hours) of administration to be investigationally related to the drug. Other adverse events could last longer, such as bruising, pain, bleeding or infection at the IV injection site. We will attempt to contact the subject at least one working day after the ^18^F-FSPG PET/CT scan, at least 12 hours after injection, regarding possible AEs relating to the study.

We have created a reporting form for tracking AEs related to radiopharmaceuticals given in “microdose” (sub-physiologic) quantity. This form will facilitate and better reflect reporting AEs related to diagnosis-only investigations of sub-physiological mass quantity radiopharmaceuticals. **This AE tracking form will be submitted as a separate document to the IRB.**

## Serious Adverse Events (SAE) are defined in accordance with the NCI’s CTCAE Ver. 4.0.

A serious adverse events is defined as, but not limited to, events that:

• result in death

• are life-threatening (The patient was at risk of death at the time of the event. It does not refer to an event which hypothetically might have caused death if it were more severe)

• requires inpatient hospitalization or prolongation of existing hospitalization

• results in persistent or significant disability/incapacity, or in a congenital anomaly/birth defect

All serious adverse events, regardless of causality to protocol-indicated treatment, will be reported to the Principal Investigator and/or the Study Coordinator at each institution, and also to the Coordinating Center.

All serious adverse events must be reported to the Coordinating Center within 24 hours of the investigator becoming aware of the event. Events should be reported using the Vanderbilt Coordinating Center SAE Form

as well as Form FDA 3500A (Mandatory Reporting Form for investigational agents). The FDA form can be found online at [https://www.accessdata.fda.gov/scripts/medwatch/](about:blank).

Both forms must be fully completed and emailed (preferred), faxed, or scanned to:

**ATTN: VICC CTSR Personnel**

**EMAIL: Coordinating.Center@vumc.org**

**FAX: (615) 875-0040**

If SAE documents are faxed, the Coordinating Center must be notified via email as well. Follow-up information must also be reported within 24 hours of receipt of the information by the investigator.

The Coordinating Center will disseminate information regarding serious adverse events to the participating sites as described in FDA guidance only in the case that the event(s) is/are unexpected, and is/are believed to be related (i.e. possibly, probably, or definitely) to the study medication. The Coordinating Center will be responsible for reporting of events to the FDA and supporters, as appropriate.

**Institutional Review Board**

All adverse events and serious adverse events will be reported to the IRB per current institutional standards. If an adverse event requires modification of the informed consent, these modifications will be provided to the IRB with the report of the adverse event. If an adverse event requires modification to the study protocol, these modifications will be provided to the IRB as soon as possible.

**10. Study Withdrawal / Discontinuation**

Patients will be withdrawn from the study for the following reasons: withdrawal of patient consent, failure to adhere to physician instructions and protocol, and if the patient’s physician considers withdrawal to be in a given patient’s best interest.

**11. Statistical Considerations**

**Study Design**

As we have shown from a large review of patients referred to us for IPNs and lung masses, ^18^F-FDG PET/CT has 92% sensitivity but 40% specificity for diagnosis of cancer, primarily due to inflammatory (especially granulomatous) lung nodules in our population. For ^18^F-FSPG to have a significant impact, we assume that the sensitivity of ^18^F-FSPG will be equal to ^18^F-FDG, but that the specificity of ^18^F-FSPG for the diagnosis of lung cancer will be 80%. Given the preliminary data we have shown with virtually no ^18^F-FSPG uptake in an active granulomatous abscess, we are very optimistic that our approach could succeed.

Our preliminary proposed investigation will include 30 subjects who had both PET scans done FDG and FSPG of patients meeting enrollment criteria and referred from the VUMC Pulmonary Medicine or Thoracic Surgical Oncology services. We will include those IPNs or lung cancers discovered *via* lung cancer screening or referred for a non-screened discovered IPN or cancer. If our preliminary data are encouraging, we will seek additional funding to enroll more to achieve statistical significance. However, we predict that we will need to extend the study to a total of 70 subjects completing a study to achieve significance. Our plans for the additional funding will be to approach the current IP holder (Piramal Life Sciences) as well as to apply for extra-mural funding with initial data from our initial subjects.

***Go/No-Go criteria:*** To verify that data collected in this pilot imaging study supports proceeding to a larger efficacy trial, clear Go/No-Go criteria, which will be assessed at the end of enrollment and data analysis, have been established for each aim, and include:

A. Aim 1 – ^18^F-FSPG PET/CT has equal sensitivity to ^18^-FDG PET/CT, with ≥50% specificity, compared to the proven 40% specificity of ^18^F-FDG PET/CT, for the diagnosis of indeterminate lung nodules, in our area with high endemic rates of fungal nodules.

B. Aim 2 – ^18^F-FSPG PET/CT is at least equivalent in overall accuracy to ^18^F-FDG PET/CT for staging of lung cancer.

C. Aim 3 –^18^F-FSPG uptake on PET/CT imaging correlates significantly with co-expressed (presumed heterodimer) CD44 and Xc- expression levels in surgical pathologic specimens; if not, pursue other explanations for ^18^F-FSPG uptake.

A positive result in Aim 1 would support additional clinical trials (Go), but failure to achieve either sufficient sensitivity or selectivity for lung cancer would contraindicate further study (No-Go) at the present time.

**Primary Endpoints: Results from ^18^F-FSPG PET/CT:**

- 1. Demonstrate significance accuracy in discrimination of benign from malignant nodules/masses equal to or greater than ^18^F-FDG PET/CT, especially for inflammatory/infectious lung nodules, or trend toward significance should additional subjects be enrolled.
  2. Demonstrate equivalent or superior accuracy for staging of lung cancer compared to ^18^F-FDG PET/CT, or trend towards significance should additional subjects be enrolled.

**Sample Size and Study Power**

Because our study population will not originate solely from a lung cancer screening trial, it will be “enriched” for high risk of malignancy. Thus, the sample size calculation primarily focuses on the power to detect the minimum clinically important difference in the specificity of ^18^F-FDG and ^18^F-FSPG tests ([19](#_ENREF_19)). Accordingly, comparing to ^18^F-FDG PET/CT scans, we hypothesize that use of the ^18^F-FSPG PET/CT will improve specificity for lung cancer diagnosis in our study population from 40% (^18^F-FDG) to 80% (^18^F-FSPG). We use McNemar’s test for paired data to evaluate the specificity for these two methods. We assume the proportion of the discordant pairs is 0.2 and 0.6. A sample size of 37 benign subjects provides 80% power to detect a 40% increase in specificity with a two-sided type 1 error = 5%. Sixty-two subjects will be recruited to take the historic lung cancer prevalence of 0.4 ([19](#_ENREF_19)) for the referral population to the pulmonary medicine or thoracic surgery services at VUMC for an IPN or lung mass. We will also apply a non-inferiority test to show that the sensitivity of ^18^F-FSPG is not worse than the ^18^F-FDG. A sample size of 25 cancer patients achieves 82% power at a 5% type 1 error using one-sided equivalence test when the sensitivity of ^18^F-FDG is 0.9 ([42](#_ENREF_42)) and the maximum allowable difference between ^18^F-FDG and ^18^F-FSPG is 0.2. Thus, we propose to enroll up to 60 subjects to allow for possible dropout such as subjects lost to follow-up or choosing to withdraw from the study before protocol completion.

**Data Analysis**

As part of our “go/no go” analysis as initial proof of principle, we plan to enroll 30 subjects initially with IPNs or newly discovered lung masses, to determine if this initial enrollment will demonstrate a trend toward significance in accuracy for diagnosis of IPNs or lung masses. This will be an initial, “proof of principle” investigation. If not, we will consider other imaging probes. However, if we do see a trend of significance, we plan on 70 subjects to complete a study.

We will not know at time of enrollment if the subject has a benign or malignant IPN or mass. Historically, we have a 40% prevalence of cancer in our referral base, which, heretofore, is not a “screened” population. Thus, with 70 subjects completing a study, we expect that, after tissue confirmation or adequate clinical follow-up to establish benignity is established, to have about 42 subjects with benign nodules and 28 with cancer. The analysis of Specific Aim 1 will focus on the ^18^F-FSPG PET/CT’s ability to discriminate up-front benign from malignant lung nodules/masses in all subjects. Analysis of Specific Aim 2 will then focus on analysis of known/confirmed lung cancers. Specific Aim 3 analysis will correlate the biomarker findings as described elsewhere in the Project Narrative with the findings of Specific Aims 1 and 2.

Sensitivity, specificity, overall accuracy and ROC curves will be generated and compared for both ^18^F-FDG and ^18^F-FSPG tests. Wilcoxon rank-sum or Kruskal-Wallis tests will be applied for the group comparisons of immunohistochemical staining (CD44 and x_C_^-^ expression levels, Specific Aim 3) and the biodistribution characteristics of ^18^F-FDG and ^18^F-FSPG tests (Specific Aims 1 & 2). The demographic information such as age, smoking status/pack-year history will be tabulated. Descriptive statistics, including means, standard deviations, and ranges for continuous parameters (uptake expressed in SUV_max_, SUV_mean_, SUV_peak_) normalized to lean body mass and body weight, and ratios of these uptake values to cardiac blood pool and normal lung. as well as percentages and frequencies for categorical parameters, will be presented.

**12. Privacy / Confidentiality**

All reasonable efforts will be made to keep patient health information private and confidential. To satisfy the reporting requirements of regulatory bodies and study sponsors, Dr. Shah and the study team will report study results as needed to the National Institutes of Health (NIH), US Food and Drug Administration (FDA), Vanderbilt Institutional Review Board (IRB), or other state and federal regulatory bodies. In reporting results for publication, all medical information will be de-identified in compliance with HIPPA regulations. Reasonable effort will also be made to keep personal information in research records private and confidential. Access to coded research data will be limited to key study personnel. Signed consent forms will be maintained in a locked file cabinet in the Vanderbilt Clinical Trials office.

**13. Follow-up and Record Retention**

Study results will be retained in the research record for at least six years after the study is completed. At that time, the research information not already in the patient’s medical record will be stored in a locked file cabinet in the Vanderbilt Clinical Trials office. Any research information in the medical record will be kept indefinitely.

Following closure of the study, each participating center will maintain a copy of all site study records in a safe and secure location. The Coordinating Center will inform the investigator at each site at such time that the records may be destroyed.

**Study Procedure Calendar (2-year follow-up by CT only needed for subjects without a definitive diagnosis of the nodule or mass by biopsy)**

|  | Year 0:  Study Initiation | Year 1 | Year 2 |
| --- | --- | --- | --- |
| Initial Visit | x |  |  |
| Consent | x |  |  |
| Finger-stick Glucose (in PET/CT lab) | x |  |  |
| Blood draw (for pregancy test for female subjects of childbearing potential only) | x |  |  |
| Chest CT Review | SOC | SOC | SOC |
| ^18^F-FSPG PET/CT | x |  |  |
| ^18^F-FDG PET/CT | SOC |  |  |
| Adverse Event Follow-up | Performed day after ^18^F-FSPG scan as described |  |  |

**14. References**

1. Siegel R, Naishadham D, Jemal A. Cancer statistics, 2013. CA Cancer J Clin. 2013;63(1):11-30. Epub 2013/01/22. doi: 10.3322/caac.21166. PubMed PMID: 23335087.

2. Hayat MJ, Howlader N, Reichman ME, Edwards BK. Cancer statistics, trends, and multiple primary cancer analyses from the Surveillance, Epidemiology, and End Results (SEER) Program. The oncologist. 2007;12(1):20-37. Epub 2007/01/18. doi: 10.1634/theoncologist.12-1-20. PubMed PMID: 17227898.

3. Humphrey LL, Deffebach M, Pappas M, Baumann C, Artis K, Mitchell JP, Zakher B, Fu R, Slatore CG. Screening for lung cancer with low-dose computed tomography: a systematic review to update the US Preventive services task force recommendation. Annals of internal medicine. 2013;159(6):411-20. Epub 2013/07/31. doi: 10.7326/0003-4819-159-6-201309170-00690. PubMed PMID: 23897166.

4. Aberle DR, Adams AM, Berg CD, Black WC, Clapp JD, Fagerstrom RM, Gareen IF, Gatsonis C, Marcus PM, Sicks JD. Reduced lung-cancer mortality with low-dose computed tomographic screening. The New England journal of medicine. 2011;365(5):395-409. Epub 2011/07/01. doi: 10.1056/NEJMoa1102873. PubMed PMID: 21714641.

5. MacMahon H, Austin JH, Gamsu G, Herold CJ, Jett JR, Naidich DP, Patz EF, Jr., Swensen SJ. Guidelines for management of small pulmonary nodules detected on CT scans: a statement from the Fleischner Society. Radiology. 2005;237(2):395-400. Epub 2005/10/26. doi: 10.1148/radiol.2372041887. PubMed PMID: 16244247.

6. Gould MK, Donington J, Lynch WR, Mazzone PJ, Midthun DE, Naidich DP, Wiener RS. Evaluation of individuals with pulmonary nodules: when is it lung cancer? Diagnosis and management of lung cancer, 3rd ed: American College of Chest Physicians evidence-based clinical practice guidelines. Chest. 2013;143(5 Suppl):e93S-120S. Epub 2013/05/10. doi: 10.1378/chest.12-2351. PubMed PMID: 23649456; PMCID: 3749714.

7. Mariotto AB, Yabroff KR, Shao Y, Feuer EJ, Brown ML. Projections of the cost of cancer care in the United States: 2010-2020. Journal of the National Cancer Institute. 2011;103(2):117-28. Epub 2011/01/14. doi: 10.1093/jnci/djq495. PubMed PMID: 21228314; PMCID: 3107566.

8. Goulart BH, Bensink ME, Mummy DG, Ramsey SD. Lung cancer screening with low-dose computed tomography: costs, national expenditures, and cost-effectiveness. Journal of the National Comprehensive Cancer Network : JNCCN. 2012;10(2):267-75. Epub 2012/02/07. PubMed PMID: 22308519.

9. Levine MS, Weiss JM, Harrell JH, Cameron TJ, Moser KM. Transthoracic needle aspiration biopsy following negative fiberoptic bronchoscopy in solitary pulmonary nodules. Chest. 1988;93(6):1152-5. Epub 1988/06/01. PubMed PMID: 3371092.

10. Wallace JM, Deutsch AL. Flexible fiberoptic bronchoscopy and percutaneous needle lung aspiration for evaluating the solitary pulmonary nodule. Chest. 1982;81(6):665-71. Epub 1982/06/01. PubMed PMID: 7075298.

11. Islam S, Walker RC. Advanced imaging (positron emission tomography and magnetic resonance imaging) and image-guided biopsy in initial staging and monitoring of therapy of lung cancer. Cancer J. 2013;19(3):208-16. doi: 10.1097/PPO.0b013e318295185f. PubMed PMID: 23708067; PMCID: PMC4834561.

12. McKee BJ, McKee AB, Flacke S, Lamb CR, Hesketh PJ, Wald C. Initial experience with a free, high-volume, low-dose CT lung cancer screening program. Journal of the American College of Radiology : JACR. 2013;10(8):586-92. Epub 2013/04/30. doi: 10.1016/j.jacr.2013.02.015. PubMed PMID: 23623708.

13. Wender R, Fontham ET, Barrera E, Jr., Colditz GA, Church TR, Ettinger DS, Etzioni R, Flowers CR, Gazelle GS, Kelsey DK, LaMonte SJ, Michaelson JS, Oeffinger KC, Shih YC, Sullivan DC, Travis W, Walter L, Wolf AM, Brawley OW, Smith RA. American Cancer Society lung cancer screening guidelines. CA Cancer J Clin. 2013;63(2):107-17. Epub 2013/01/15. doi: 10.3322/caac.21172. PubMed PMID: 23315954; PMCID: 3632634.

14. CMS.gov. Proposed Decision Memo for Screening for Lung Cancer with Low Dose Computed Tomography (LDCT) (CAG-00439N) 2014 [cited 2014 December 19]. Available from: [http://www.cms.gov/medicare-coverage-database/details/nca-proposed-decision-memo.aspx?NCAId=274](about:blank).

15. Ma J, Ward EM, Smith R, Jemal A. Annual number of lung cancer deaths potentially avertable by screening in the United States. Cancer. 2013;119(7):1381-5. Epub 2013/02/27. doi: 10.1002/cncr.27813. PubMed PMID: 23440730.

16. Lowe VJ, Fletcher JW, Gobar L, Lawson M, Kirchner P, Valk P, Karis J, Hubner K, Delbeke D, Heiberg EV, Patz EF, Coleman RE. Prospective investigation of positron emission tomography in lung nodules. Journal of clinical oncology : official journal of the American Society of Clinical Oncology. 1998;16(3):1075-84. Epub 1998/03/21. PubMed PMID: 9508193.

17. Kelly RF, Tran T, Holmstrom A, Murar J, Segurola RJ, Jr. Accuracy and cost-effectiveness of [18F]-2-fluoro-deoxy-D-glucose-positron emission tomography scan in potentially resectable non-small cell lung cancer. Chest. 2004;125(4):1413-23. Epub 2004/04/14. PubMed PMID: 15078754.

18. Nomori H, Watanabe K, Ohtsuka T, Naruke T, Suemasu K, Uno K. Evaluation of F-18 fluorodeoxyglucose (FDG) PET scanning for pulmonary nodules less than 3 cm in diameter, with special reference to the CT images. Lung Cancer. 2004;45(1):19-27. Epub 2004/06/16. doi: 10.1016/j.lungcan.2004.01.009. PubMed PMID: 15196730.

19. Deppen S, Putnam JB, Jr., Andrade G, Speroff T, Nesbitt JC, Lambright ES, Massion PP, Walker R, Grogan EL. Accuracy of FDG-PET to diagnose lung cancer in a region of endemic granulomatous disease. Ann Thorac Surg. 2011;92(2):428-32; discussion 33. doi: 10.1016/j.athoracsur.2011.02.052. PubMed PMID: 21592456; PMCID: 3186439.

20. Croft DR, Trapp J, Kernstine K, Kirchner P, Mullan B, Galvin J, Peterson MW, Gross T, McLennan G, Kern JA. FDG-PET imaging and the diagnosis of non-small cell lung cancer in a region of high histoplasmosis prevalence. Lung Cancer. 2002;36(3):297-301. Epub 2002/05/16. PubMed PMID: 12009241.

21. Kubota R, Yamada S, Kubota K, Ishiwata K, Tamahashi N, Ido T. Intratumoral distribution of fluorine-18-fluorodeoxyglucose in vivo: high accumulation in macrophages and granulation tissues studied by microautoradiography. Journal of nuclear medicine : official publication, Society of Nuclear Medicine. 1992;33(11):1972-80. Epub 1992/11/01. PubMed PMID: 1432158.

22. Vander Heiden MG, Cantley LC, Thompson CB. Understanding the Warburg effect: the metabolic requirements of cell proliferation. Science. 2009;324(5930):1029-33. Epub 2009/05/23. doi: 324/5930/1029 [pii]

10.1126/science.1160809. PubMed PMID: 19460998.

23. Patel VK, Naik SK, Naidich DP, Travis WD, Weingarten JA, Lazzaro R, Gutterman DD, Wentowski C, Grosu HB, Raoof S. A practical algorithmic approach to the diagnosis and management of solitary pulmonary nodules: part 1: radiologic characteristics and imaging modalities. Chest. 2013;143(3):825-39. doi: 10.1378/chest.12-0960. PubMed PMID: 23460160.

24. Patel VK, Naik SK, Naidich DP, Travis WD, Weingarten JA, Lazzaro R, Gutterman DD, Wentowski C, Grosu HB, Raoof S. A practical algorithmic approach to the diagnosis and management of solitary pulmonary nodules: part 2: pretest probability and algorithm. Chest. 2013;143(3):840-6. doi: 10.1378/chest.12-1487. PubMed PMID: 23460161.

25. McFall-Ngai M, Hadfield MG, Bosch TC, Carey HV, Domazet-Loso T, Douglas AE, Dubilier N, Eberl G, Fukami T, Gilbert SF, Hentschel U, King N, Kjelleberg S, Knoll AH, Kremer N, Mazmanian SK, Metcalf JL, Nealson K, Pierce NE, Rawls JF, Reid A, Ruby EG, Rumpho M, Sanders JG, Tautz D, Wernegreen JJ. Animals in a bacterial world, a new imperative for the life sciences. Proc Natl Acad Sci U S A. 2013. doi: 10.1073/pnas.1218525110. PubMed PMID: 23391737.

26. Koglin N, Mueller A, Berndt M, Schmitt-Willich H, Toschi L, Stephens AW, Gekeler V, Friebe M, Dinkelborg LM. Specific PET imaging of xC- transporter activity using a 18F-labeled glutamate derivative reveals a dominant pathway in tumor metabolism. Clinical cancer research : an official journal of the American Association for Cancer Research. 2011;17(18):6000-60011. Epub 2011/07/14. doi: 1078-0432.CCR-11-0687 [pii]

10.1158/1078-0432.CCR-11-0687. PubMed PMID: 21750203.

27. Herrmann K, Buck AK, Schuster T, Junger A, H AW, Graf N, Ringshausen I, Rudelius M, Wester HJ, Schwaiger M, Keller U, Dechow T. Predictive Value of Initial 18F-FLT Uptake in Patients with Aggressive Non-Hodgkin Lymphoma Receiving R-CHOP Treatment. Journal of nuclear medicine : official publication, Society of Nuclear Medicine. 2011;52(5):690-6. Epub 2011/04/19. doi: 10.2967/jnumed.110.084566. PubMed PMID: 21498532.

28. Lewerenz J, Hewett SJ, Huang Y, Lambros M, Gout PW, Kalivas PW, Massie A, Smolders I, Methner A, Pergande M, Smith SB, Ganapathy V, Maher P. The cystine/glutamate antiporter system x(c)(-) in health and disease: from molecular mechanisms to novel therapeutic opportunities. Antioxidants & redox signaling. 2013;18(5):522-55. Epub 2012/06/07. doi: 10.1089/ars.2011.4391. PubMed PMID: 22667998; PMCID: 3545354.

29. Lo M, Wang YZ, Gout PW. The x(c)- cystine/glutamate antiporter: a potential target for therapy of cancer and other diseases. Journal of cellular physiology. 2008;215(3):593-602. Epub 2008/01/09. doi: 10.1002/jcp.21366. PubMed PMID: 18181196.

30. Chae SY, Choi CM, Shim TS, Park Y, Park CS, Lee HS, Lee SJ, Oh SJ, Kim SY, Baek S, Koglin N, Stephens AW, Dinkelborg LM, Moon DH. Exploratory Clinical Investigation of (4S)-4-(3-18F-Fluoropropyl)-l-Glutamate PET of Inflammatory and Infectious Lesions. J Nucl Med. 2016;57(1):67-9. doi: 10.2967/jnumed.115.164020. PubMed PMID: 26471694.

31. Baek S, Choi CM, Ahn SH, Lee JW, Gong G, Ryu JS, Oh SJ, Bacher-Stier C, Fels L, Koglin N, Hultsch C, Schatz CA, Dinkelborg LM, Mittra ES, Gambhir SS, Moon DH. Exploratory clinical trial of (4S)-4-(3-[18F]fluoropropyl)-L-glutamate for imaging xC- transporter using positron emission tomography in patients with non-small cell lung or breast cancer. Clin Cancer Res. 2012;18(19):5427-37. doi: 10.1158/1078-0432.CCR-12-0214. PubMed PMID: 22893629.

32. Tian J, Yang X, Yu L, Chen P, Xin J, Ma L, Feng H, Tan Y, Zhao Z, Wu W. A multicenter clinical trial on the diagnostic value of dual-tracer PET/CT in pulmonary lesions using 3'-deoxy-3'-18F-fluorothymidine and 18F-FDG. J Nucl Med. 2008;49(2):186-94. Epub 2008/01/18. doi: jnumed.107.044966 [pii]

10.2967/jnumed.107.044966. PubMed PMID: 18199618.

33. Hustinx R, Lemaire C, Jerusalem G, Moreau P, Cataldo D, Duysinx B, Aerts J, Fassotte MF, Foidart J, Luxen A. Whole-body tumor imaging using PET and 2-18F-fluoro-L-tyrosine: preliminary evaluation and comparison with 18F-FDG. Journal of nuclear medicine : official publication, Society of Nuclear Medicine. 2003;44(4):533-9. Epub 2003/04/08. PubMed PMID: 12679396.

34. Beer AJ, Lorenzen S, Metz S, Herrmann K, Watzlowik P, Wester HJ, Peschel C, Lordick F, Schwaiger M. Comparison of integrin alphaVbeta3 expression and glucose metabolism in primary and metastatic lesions in cancer patients: a PET study using 18F-galacto-RGD and 18F-FDG. Journal of nuclear medicine : official publication, Society of Nuclear Medicine. 2008;49(1):22-9. Epub 2007/12/14. doi: 10.2967/jnumed.107.045864. PubMed PMID: 18077538.

35. Baek S, Mueller A, Lim YS, Lee HC, Lee YJ, Gong G, Kim JS, Ryu JS, Oh SJ, Lee SJ, Bacher-Stier C, Fels L, Koglin N, Schatz CA, Dinkelborg LM, Moon DH. (4S)-4-(3-18F-fluoropropyl)-L-glutamate for imaging of xC transporter activity in hepatocellular carcinoma using PET: preclinical and exploratory clinical studies. J Nucl Med. 2013;54(1):117-23. doi: 10.2967/jnumed.112.108704. PubMed PMID: 23232273.

36. Lieberman BP, Ploessl K, Wang L, Qu W, Zha Z, Wise DR, Chodosh LA, Belka G, Thompson CB, Kung HF. PET imaging of glutaminolysis in tumors by 18F-(2S,4R)4-fluoroglutamine. J Nucl Med. 2011;52(12):1947-55. doi: 10.2967/jnumed.111.093815. PubMed PMID: 22095958.

37. Koglin N, Mueller A, Berndt M, Schmitt-Willich H, Toschi L, Stephens AW, Gekeler V, Friebe M, Dinkelborg LM. Specific PET imaging of xC- transporter activity using a (1)(8)F-labeled glutamate derivative reveals a dominant pathway in tumor metabolism. Clin Cancer Res. 2011;17(18):6000-11. doi: 10.1158/1078-0432.CCR-11-0687. PubMed PMID: 21750203.

38. Deppen SA, Blume JD, Kensinger CD, Morgan AM, Aldrich MC, Massion PP, Walker RC, McPheeters ML, Putnam JB, Jr., Grogan EL. Accuracy of FDG-PET to diagnose lung cancer in areas with infectious lung disease: a meta-analysis. JAMA. 2014;312(12):1227-36. doi: 10.1001/jama.2014.11488. PubMed PMID: 25247519.

39. Henschke CI, Yip R, Yankelevitz DF, Smith JP. Definition of a positive test result in computed tomography screening for lung cancer: a cohort study. Annals of internal medicine. 2013;158(4):246-52. Epub 2013/02/20. doi: 10.7326/0003-4819-158-4-201302190-00004. PubMed PMID: 23420233.

40. Hassanein M, Hoeksema MD, Shiota M, Qian J, Harris BK, Chen H, Clark JE, Alborn WE, Eisenberg R, Massion PP. SLC1A5 mediates glutamine transport required for lung cancer cell growth and survival. Clinical cancer research : an official journal of the American Association for Cancer Research. 2013;19(3):560-70. Epub 2012/12/06. doi: 10.1158/1078-0432.CCR-12-2334. PubMed PMID: 23213057; PMCID: 3697078.

41. Kikuchi T, Hassanein M, Amann JM, Liu Q, Slebos RJ, Rahman SM, Kaufman JM, Zhang X, Hoeksema MD, Harris BK, Li M, Shyr Y, Gonzalez AL, Zimmerman LJ, Liebler DC, Massion PP, Carbone DP. In-depth proteomic analysis of nonsmall cell lung cancer to discover molecular targets and candidate biomarkers. Mol Cell Proteomics. 2012;11(10):916-32. doi: 10.1074/mcp.M111.015370. PubMed PMID: 22761400; PMCID: 3494148.

42. Kinsinger LS, Atkins D, Provenzale D, Anderson C, Petzel R. Implementation of a New Screening Recommendation in Health Care: The Veterans Health Administration's Approach to Lung Cancer Screening. Ann Intern Med. 2014;161(8):597-8. doi: 10.7326/M14-1070. PubMed PMID: 25111673.
